# SKI complex loss renders 9p21.3-deleted or MSI-H cancers dependent on PELO

**DOI:** 10.1038/s41586-024-08509-3

**Published:** 2025-02-05

**Authors:** Patricia C. Borck, Isabella Boyle, Kristina Jankovic, Nolan Bick, Kyla Foster, Anthony C. Lau, Lucy I. Parker-Burns, Daniel A. Lubicki, Tianxia Li, Ashir A. Borah, Nicholas J. Lofaso, Sohani Das Sharma, Tessla Chan, Riya V. Kishen, Anisah Adeagbo, Srivatsan Raghavan, Elisa Aquilanti, John R. Prensner, J. Michael Krill-Burger, Todd R. Golub, Catarina D. Campbell, Joshua M. Dempster, Edmond M. Chan, Francisca Vazquez

**Affiliations:** 1 Broad Institute of MIT and Harvard, Cambridge, MA USA; 2 Herbert Irving Comprehensive Cancer Center, Columbia University Irving Medical Center, New York, NY USA; 3 Department of Medical Oncology, Dana Farber Cancer Institute, Harvard Medical School, Boston, MA USA; 4 Division of Neuro-Oncology, Department of Medical Oncology, Dana Farber Cancer Institute, Harvard Medical School, Boston, MA USA; 5 Department of Pediatrics and Biological Chemistry, Division of Hematology/Oncology, University of Michigan, Ann Arbor, MI USA; 6 Department of Pediatric Oncology, Dana Farber Cancer Institute, Harvard Medical School, Boston, MA USA; 7 Department of Medicine, Division of Hematology/Oncology, Vagelos College of Physicians and Surgeons, Columbia University Irving Medical Center, New York, NY USA; 8 New York Genome Center, New York, NY USA

**Keywords:** Targeted therapies, Predictive markers

## Abstract

Cancer genome alterations often lead to vulnerabilities that can be used to selectively target cancer cells. Various inhibitors of such synthetic lethal targets have been approved by the FDA or are in clinical trials, highlighting the potential of this approach^1–3^. Here we analysed large-scale CRISPR knockout screening data from the Cancer Dependency Map and identified a new synthetic lethal target, *PELO*, for two independent molecular subtypes of cancer: biallelic deletion of chromosomal region 9p21.3 or microsatellite instability-high (MSI-H). In 9p21.3-deleted cancers, *PELO* dependency emerges from biallelic deletion of the 9p21.3 gene *FOCAD*, a stabilizer of the superkiller complex (SKIc). In MSI-H cancers, PELO is required owing to MSI-H-associated mutations in *TTC37* (also known as *SKIC3*), a critical component of the SKIc. We show that both cancer subtypes converge to destabilize the SKIc, which extracts mRNA from stalled ribosomes. In SKIc-deficient cells, PELO depletion induces the unfolded protein response, a stress response to accumulation of misfolded or unfolded nascent polypeptides. Together, our findings indicate *PELO* as a promising therapeutic target for a large patient population with cancers characterized as MSI-H with deleterious *TTC37* mutations or with biallelic 9p21.3 deletions involving *FOCAD*.

## Abstract (web summary)

Analysis of large-scale CRISPR screening data, combined with experiments in patient-derived tumour organoid models, identifies *PELO* as a potential therapeutic target in chromosomal 9p21.3-deleted cancers and microsatellite-unstable cancers harbouring specific mutations.

## Main

**Fig. 1:**
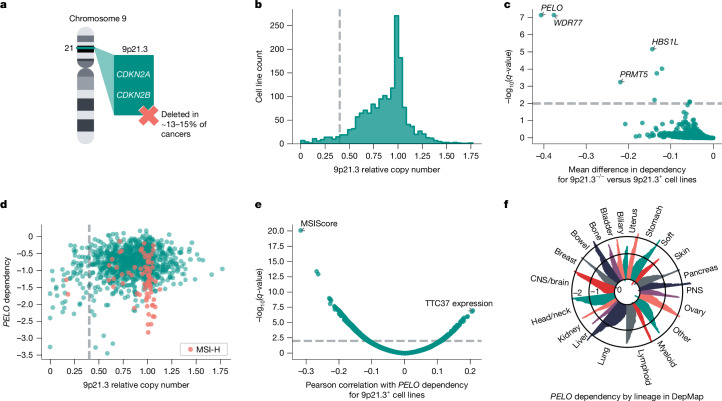
Analyses of DepMap data to identify dependencies associated with 9p21.3 deletion. **a**, Illustration of chromosome 9 cytoband 9p21.3, which is biallelically deleted (9p21.3^−/−^) in approximately 13–15% of human cancers. **b**, Histogram of 9p21.3 relative copy number across DepMap cell lines (*n* = 1,750). Threshold of less than 0.4 (dashed line) indicates cell lines characterized as 9p21.3^−/−^. **c**, *q*-values from left-tailed Student’s *t*-test between 9p21.3^−/−^ (less than 0.4 threshold, *n* = 63 cell lines) or at least one intact copy of 9p21.3 (9p21.3^+^, *n* = 1,037 cell lines) plotted against the mean difference in gene dependency in DepMap. **d**, *PELO* dependency plotted against 9p21.3 relative copy number (*n* = 1,100 cell lines), with MSI-H cell lines (MSIsensor2 MSIScore > 20, *n* = 73) highlighted. **e**, Univariate associations of DepMap omics data with *PELO* dependency score in 9p21.3^+^ cell lines (*n* = 1,037). **f**, *PELO* dependency score for cell lines (*n* = 1,100) grouped by OncoTree lineage. Ovary, ovary and/or fallopian tube; bladder: bladder and/or urinary tract; soft, soft tissue; stomach, oesophagus and/or stomach; head/neck, head and neck; PNS, peripheral nervous system; biliary, biliary tract; ampulla, ampulla of Vater. Relative copy number, gene dependency and omics data are from DepMap 23Q4 or 24Q2 release. Source Data

Homozygous deletion of chromosomal region 9p21.3 is among the most frequently observed somatic copy number alterations in human cancers, occurring in approximately 13–15% of all cancers^4,5^ (Fig. 1a). Many 9p21.3-loss cancers are associated with poor clinical outcomes, including subsets of glioblastoma, urothelial carcinoma of the bladder, pancreatic ductal adenocarcinoma, oesophageal adenocarcinoma and non-small-cell lung cancer^6–11^. Loss of tumour suppressors *CDKN2A* or *CDKN2AB* is thought to be a key driver of 9p21.3 deletions (9p21.3^−/−^) in cancers. However, recent studies suggest that deletions of neighbouring genes are also important for oncogenesis, which might explain why this chromosomal region is deleted rather than isolated inactivation of *CDKN2A* or *CDKN2B* *MTAP*, which lies adjacent to *CDKN2A*, is a putative tumour suppressor gene that is frequently codeleted with *CDKN2A* and *CDKN2B*^12,13^. Studies have also linked the loss of an interferon gene cluster on 9p21.3 to immune evasion and primary resistance to immune checkpoint inhibitors^5,14^.

Large deletions of chromosomal regions can provide opportunities for cancer treatment. In particular, codeletion of passenger genes in proximity to tumour suppressors can lead to collateral lethality and create potential vulnerabilities specific to those cancers^15–18^. These codeleted genes may have critical roles in cell maintenance, and their loss can lead to newly acquired dependencies on related or paralogous genes. For instance, large-scale RNA interference data from the Cancer Dependency Map (DepMap) and Project Drive led to the discovery of PRMT5 as a promising target in 9p21.3^−/−^ cancers through its synthetic lethal interactions with *MTAP* deletion^19,20^. Although clinical trials of PRMT5 inhibitors and related MAT2A inhibitors for patients for *MTAP*-deleted cancers are underway, the frequency, poor outcomes and immune checkpoint inhibitor resistance associated with 9p21.3^−/−^ cancers highlight an urgent need for further therapeutic strategies for this broad class of cancers^21,22^.

**Extended Data Fig. 1:**
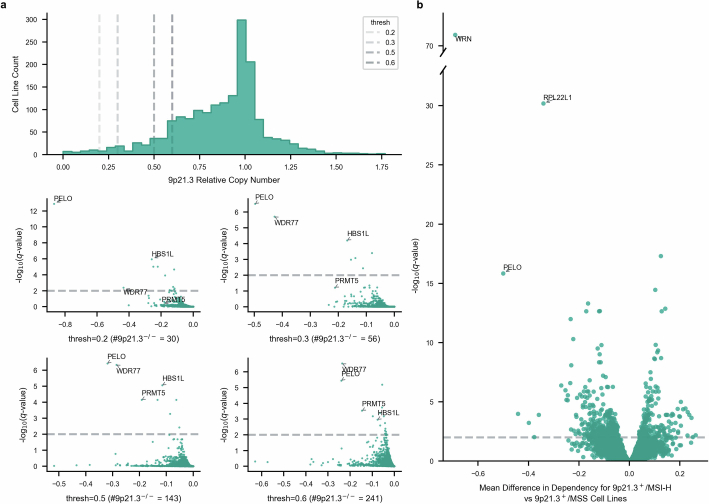
Analyses of functional genomic datasets identify *PELO* as preferential dependency. (**a**) Upper figure: histogram of 9p21.3 relative copy number across cell lines. Dashed vertical lines represent 9p21.3 relative copy number thresholds between 0.2 and 0.6 tested for calling 9p21.3^−/−^. Lower figures: *q*-value from left-tailed Student’s t-test plotted against the mean difference of gene dependency scores between 9p21.3^−/−^ or 9p21.3^+^cell lines for each indicated threshold. (**b**) *q*-value from two-tailed Student’s t-test plotted against the mean difference of dependency scores between 9p21.3^+^/MSI-H (*n* = 71) and 9p21.3^+^/MSS cell lines (*n* = 966). Source Data

Here, we used large-scale CRISPR knockout screening datasets from DepMap to identify further synthetic lethal targets associated with 9p21.3^−/−^ in cancer^23^. Using data from the DepMap 23Q4 release, we first computed the relative copy number for 9p21.3 for each cell line using a weighted average across the cytoband^24^ (Fig. 1b). We tested several thresholds for calling 9p21.3 loss and performed left-tailed Student’s *t*-tests comparing CRISPR gene effect scores for cell lines above or below each 9p21.3 relative copy number threshold (Fig. 1b and Extended Data Fig. 1a). Each analysis using a 9p21.3 relative copy number threshold of 0.5 or less identified *PELO* as the top preferential dependency in cell lines with low relative 9p21.3 copy number (effect size = −0.406, *n* = 1,100, *q* = 7.58 × 10^−8^; Fig. 1c and Extended Data Fig. 1a). We also observed that cell lines with greater loss of 9p21.3 were more likely to be preferentially dependent on *PELO* for viability. For the remainder of our analyses, we used a relative copy number threshold of 0.4 to distinguish models with 9p21.3^−/−^ (less than 0.4, *n* = 63) versus models with at least one intact copy of 9p21.3 (9p21.3^+^, greater than or equal to 0.4, *n* = 1,037) (Fig. 1b). Our analysis also identified *HBS1L* as a preferential vulnerability in 9p21.3^−/−^ cell lines (left-tailed Student’s *t*-test, effect size = −0.144, *n* = 1,100, *q* = 7.10 × 10^−6^). As PELO interacts with HBS1L to recruit ACBE1 to promote dissociation of stalled ribosomes and release intact peptidyl-transfer RNA, this observation supported our hypothesis that *PELO* is preferentially required in 9p21.3^−/−^ cancers^25,26^ (Fig. 1c and Extended Data Fig. 1a). As expected, our analyses also recovered the PRMT5–WDR77 complex, a previously described synthetic lethal dependency with loss of the 9p21.3 gene *MTAP*, for nearly all thresholds tested^19,27^ (left-tailed Student’s *t*-test for threshold of 0.4; *PRMT5*: effect size = −0.220, *n* = 1,100, *q* = 5.76 × 10^−4^; *WDR77*: effect size = −0.377, *n* = 1,100, *q* = 7.54 × 10^−8^; Fig. 1c and Extended Data Fig. 1a).

We observed that some 9p21.3^+^ cell lines were dependent on *PELO* for survival (Fig. 1d). To determine whether there were any other genomic features associated with *PELO* dependency, we analysed DepMap expression, copy number, mutation and genomic signature data for correlation with *PELO* dependency. According to this analysis, the MSIsensor2 MSIScore, a measure of MSI from next-generation DNA sequencing, was the top correlated feature associated with *PELO* dependency in 9p21.3^+^ cells^28^ (Pearson’s *R* = −0.316, *n* = 1037, *q* = 9.52 × 10^−21^; Fig. 1e). MSI-H (MSIsensor2 score > 20) is a hypermutable state observed in substantial subsets of colon, endometrial, gastric and ovarian cancers^9,29,30^. We next compared 9p21.3^+^/MSI-H and 9p21.3^+^/microsatellite stable (MSS) cell lines and found that *PELO* scored as a strong preferential dependency in MSI-H cell lines, second only to the previously described synthetic lethal target Werner helicase^31,32^ (two-tailed Student’s t-test; *PELO*: effect size = −0.500, *n* = 1037, *q* = 1.45 × 10^−16^; Werner helicase: effect size = −0.691, *n* = 1037, *q* = 2.02 × 10^−72^; Extended Data Fig. 1b). *PELO* dependency was highly variable across many cancer lineages, with those associated with MSI-H or 9p21.3^−/−^ enriched for strong *PELO* dependency (Fig. 1f). Collectively, these data raise the hypothesis that cancers with one of two predictive biomarkers, biallelic large 9p21.3 deletions or MSI-H, preferentially require *PELO* for survival.

**Fig. 2:**
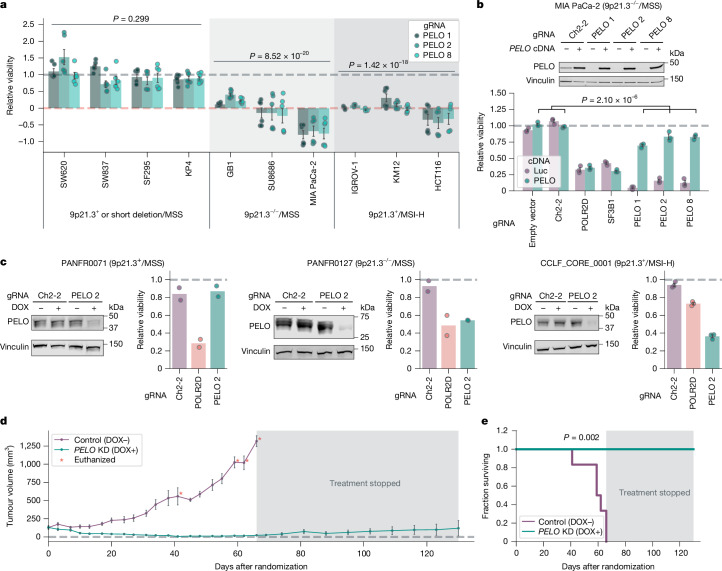
Focused validation of *PELO* dependency in vitro and in vivo. **a**, Viability effect of CRISPRi-mediated *PELO* knockdown (KD) normalized to average of negative controls (grey dashed line; empty vector and/or gRNA Ch2-2) and positive controls (orange dotted line; gRNA for *SF3B1* and/or *POLR2D*) for 9p21.3^+^ or short deletion/MSS (*n* = 4), 9p21.3^−/−^/MSS (*n* = 3) and 9p21.3^+^/MSI-H cell lines (*n* = 3). **b**, Top, immunoblots of PELO and vinculin in MIA PaCa-2 cells transduced with the indicated gRNA ± *PELO* cDNA. Bottom, viability scores normalized to average viability of negative controls. **c**, Immunoblots of PELO and vinculin levels in the indicated patient-derived tumour organoid models. Relative viability following DOX-induced knockdown of the indicated gRNA, relative to DMSO control. **d**, Average tumour volume over time for nude mice with subcutaneous engraftment of MIA PaCa-2 cells following randomization to a standard diet (control) or DOX-containing diet to induce *PELO* knockdown. **e**, Kaplan–Meier survival plot for mice randomized to DOX-containing (*n* = 5) or standard (*n* = 6) diet. Data are mean ± s.e.m. of biological replicates: *n* = 6 for all cell lines except KP4 and GB1, for which *n* = 7, and IGROV-1, for which *n* = 3 in **a**, *n* = 3 in **b**, *n* = 2 for PANFR0127 and PANFR0071 and *n* = 3 for CCLF_CORE_0001 in **c**; *n* = 9 tumours for standard diet and *n* = 10 for DOX-containing diet in **d**. Significance was calculated as follows: left-tailed Student’s *t*-test (**a**), right-tailed Student’s *t*-test (**b**), pairwise log-rank test (**e**). Representative data are shown from two experiments in **a** and one experiment in **b**–**e**. Experiments were performed twice for **a**–**c** and once for **d** and **e**. For immunoblots, vinculin was used as loading control. Source Data

**Extended Data Fig. 2:**
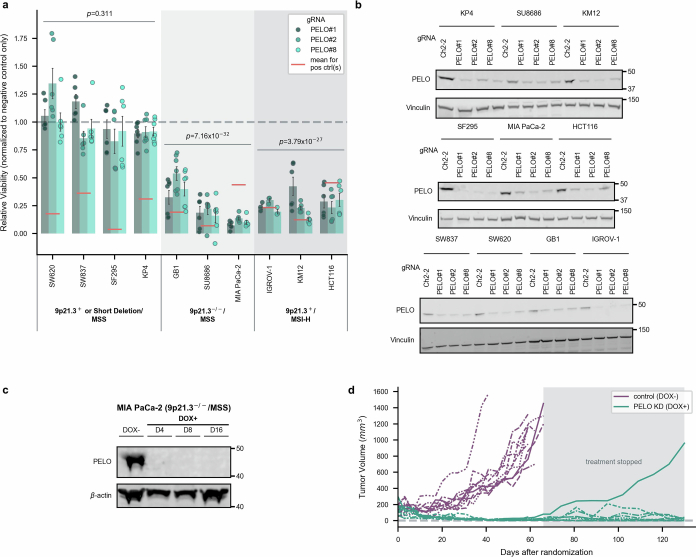
Focused validation of *PELO* dependency. (**a**) Relative viability normalizing to negative control, with mean of positive controls per cell line represented by horizontal red lines. (**b**) Immunoblot of PELO and Vinculin levels in indicated cell lines 4-6 days following transduction of indicated gRNAs. (**c**) Immunoblot of PELO and β-actin levels in engrafted MIA PaCa-2 tumors *n* = 1 mice on standard diet (DOX−) or *n* = 3 mice on DOX-containing diet (DOX+). DOX+ tumors were harvested at indicated times following the start of the diet. (**d**) Tumor volume plotted by time following randomization for individual MIA PaCa-2 xenograft growth ± DOX-induced *PELO* KD, showing all tumors (*n* = 19). All DOX-treated mice resumed a standard diet 66 days following randomization. Data are mean ± s.e.m. of biological replicates: *n* = 6 for all cell lines except KP4 and GB1 where *n* = 7 and IGROV-1 where *n* = 3 in (**a**), and *n* = 9 tumors on standard diet and *n* = 10 for DOX-containing diet in (**d**). *n* = 4 mice for (**c**). Significance was calculated as follows: (**a**) left-tailed Student’s t-test. Representative data from two experiments in (**a,b**) and one experiment in (**c,d**). Experiments were performed twice for (**a,b**) and once for (**c,d**). For immunoblots, Vinculin and β-actin were used as loading controls. Source Data

As a first step of validation, we interrogated the viability effects of knocking down *PELO* in ten different cell lines with CRISPR interference (CRISPRi). These cell lines consisted of a panel representing 9p21.3^+^/MSS (two cell lines), 9p21.3^−/−^/MSS (three cell lines) or 9p21.3^+^/MSI-H (three cell lines). In addition, we included SF295 and KP4, cell lines that harbour short deletions in 9p21.3, although they were scored as 9p21.3^+^ according to our criteria. We used dCas9–KRAB, a catalytically dead Cas9 fused to the transcriptional repressor KRAB, and three different CRISPRi guide RNAs (gRNA 1, 2 and 3) to knock down *PELO*^33^. *PELO* knockdown strongly impaired the viability of 9p21.3^−/−^/MSS (left-tailed Student’s *t*-test, effect size = −1.20, *n* = 85, *P* = 8.52 × 10^−20^) and 9p21.3^+^/MSI-H (left-tailed Student’s *t*-test, effect size = −1.10, *n* = 66, *P* = 1.42 × 10^−18^; Fig. 2a and Extended Data Fig. 2a,b) cell lines, approximating the effects of pan-essential control gRNAs targeting *SF3B1* or *POLR2D*. By contrast, for 9p21.3^+^/MSS or 9p21.3 short deletion/MSS cell lines, *PELO* knockdown showed no significant difference in terms of effect on viability compared with negative control gRNAs targeting an intergenic region on chromosome 2 (Ch2-2) and/or an empty vector control (left-tailed Student’s *t*-test, effect size = −0.03, *n* = 112, *P* = 0.299). These results provide orthogonal data that *PELO* is a preferential dependency in cell lines with large deletions of 9p21.3 and MSI-H cell lines.

To confirm that these viability effects were attributable to *PELO* loss, we studied whether exogenous expression of *PELO* could rescue cells from CRISPRi-mediated suppression of endogenous *PELO*. We found that *PELO* cDNA expression from an *EEF1A1* promoter was not affected by the three CRISPRi gRNAs targeting endogenous *PELO* in MIA PaCa-2 (9p21.3^−/−^MSS) cells. Correspondingly, *PELO* cDNA expression rescued MIA PaCa-2 cells from the viability effects of gRNAs targeting *PELO*, indicating that CRISPRi-associated impairment of viability was due to on-target *PELO* knockdown (right-tailed Student’s *t*-test, effect size = 0.675, *n* = 6, *P* = 2.10 × 10^−6^ ; Fig. 2b).

We next sought to validate *PELO* dependency in tumour organoid models for which we did not have large-scale CRISPR data and for which we could therefore only predict any requirement for *PELO* on the basis of the two identified biomarkers. Specifically, we interrogated the viability effects of *PELO* depletion in three patient-derived organoid models representing a 9p21.3^+^/MSS pancreatic cancer (PANFR0071), 9p21.3^−/−^/MSS pancreatic cancer (PANFR0127) and an 9p21.3^+^/MSI-H colorectal cancer (CCLF_CORE_0001). Using CRISPR repressor dCas9–KRAB–MeCP2 with a doxycycline (DOX)-inducible gRNA, we evaluated the effects of DOX-inducible gRNAs targeting Ch2-2, *POLR2D* or *PELO*^34^. We first confirmed that DOX treatment suppressed PELO protein levels in cells with DOX-inducible *PELO* gRNA 2 (Fig. 2c). *PELO* depletion substantially impaired the viability of 9p21.3^−/−^and MSI-H models, to a level similar to that observed with *POLR2D* knockdown (Fig. 2c). By contrast, *PELO* depletion in the 9p21.3^+^/MSS model had no discernible effect on viability, whereas *POLR2D* depletion still suppressed viability. These data support *PELO* as a synthetic lethal target in cells with MSI-H or large 9p21.3 deletions.

Next, we sought to evaluate the effects of PELO in tumour maintenance in vivo. We used the DOX-inducible CRISPRi system to suppress *PELO* in MIA PaCa-2 cells (9p21.3^−/−^/MSS). We subcutaneously engrafted engineered MIA PaCa-2 cells in the flanks of nude mice. Once the tumours had reached a volume of approximately 150 mm^3^ (range 50–300 mm^3^), mice were randomized to a DOX-containing diet or maintained on a standard diet. We verified by immunoblotting that *PELO* was knocked down as early as 4 days following transition to a DOX-containing diet (Extended Data Fig. 2c). All mice randomized to a standard diet were euthanized within 66 days owing to tumour growth reaching size end points. By contrast, we observed tumour regression in all mice treated with DOX (Fig. 2d and Extended Data Fig. 2d). After all mice in the standard diet cohort had been euthanized, mice in the DOX-treated cohort were reverted to a standard diet to determine whether previously treated tumours would regrow. By 64 days following removal of DOX, we observed that eight of nine tumours remained unchanged or impalpable, suggesting that *PELO* suppression eradicated most tumour cells. Notably, all mice randomized to DOX were alive 130 days following randomization (including 64 days without DOX), underscoring the sensitivity of these tumours to *PELO* depletion and showing significant improvement in overall survival (pairwise log-rank test, statistic = 9.71, *n* = 11, *P* = 0.002; Fig. 2e).

**Fig. 3:**
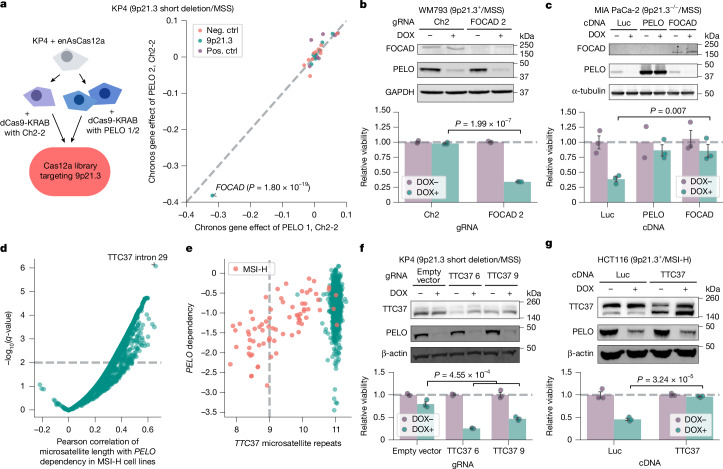
Dissecting the molecular mechanisms underlying *PELO* dependency in 9p21.3^−/−^ and MSI-H cancers. **a**, Left, CRISPR knockout (KO) screen targeting 9p21.3 genes in KP4 cells with CRISPRi knockdown using Ch2-2 gRNA (control), or *PELO* gRNA 1 or 2. Right, differences in Chronos gene effect scores between *PELO* and Ch2-2 knockdown for both *PELO* gRNAs. **b**, Top, immunoblots of FOCAD, PELO and GAPDH in WM793 cells. Bottom, relative viability of WM793 cells with Ch2 gRNA or *FOCAD* knockout ± DOX-induced *PELO* knockdown. **c**, Top, immunoblots of FOCAD, PELO and α-tubulin in MIA PaCa-2 cells. Bottom, relative viability in MIA PaCa-2 cells stably expressing indicated cDNA ± DOX-induced *PELO* knockdown. **d**, Pearson correlation of microsatellite site length with *PELO* dependency scores in MSI-H cells (*n* = 73 cell lines). **e**, Cell lines (*n* = 1,100) plotted by *PELO* dependency and length of *TTC37* intron 29 microsatellite repeats. **f**, Top, immunoblots of TTC37, PELO and β-actin in KP4 cells. Bottom, relative viability of KP4 ± *TTC37* knockout ± DOX-induced *PELO* knockdown cells. **g**, Top, immunoblots of TTC37, PELO and β-actin in HCT116. Bottom, relative viability of HCT116 cells stably expressing the indicated cDNA ± DOX-induced *PELO* knockdown. Data are mean ± s.e.m. of biological replicates: *n* = 2 in **a**; *n* = 3 in **b**, **f** and **g**; and *n* = 3 except for *PELO* cDNA in DOX− condition, for which *n* = 2, in **c**. Significance was calculated as follows: in **a**, left-tailed Wilcoxon rank-sum test; in **b** and **f**, left-tailed Student’s *t*-test; in **c** and **g**, right-tailed Student’s *t*-test. Representative data from one experiment are shown. All experiments were performed twice, except for the experiment in **a**, which was performed once. For immunoblots, GAPDH, α-tubulin and β-actin were used as loading controls. Neg. ctrl, negative control; Pos. ctrl, positive control. Source Data

**Extended Data Fig. 3:**
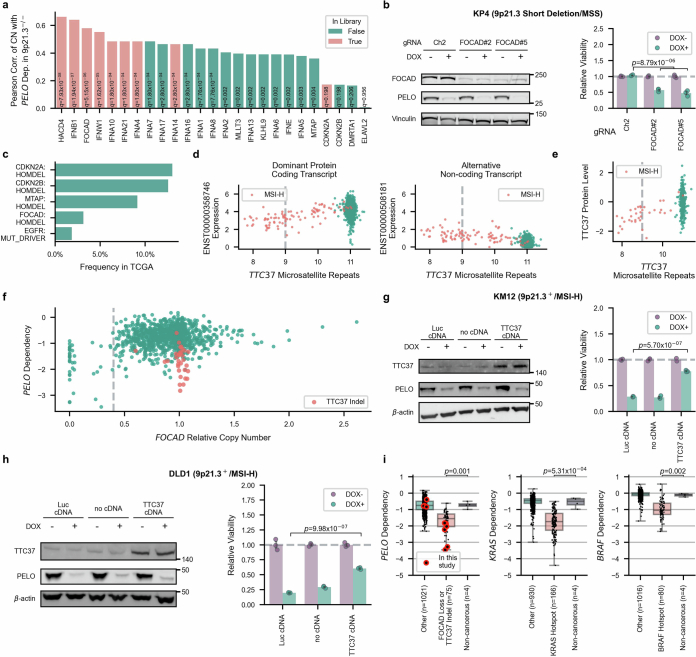
Characterizing the molecular alterations underlying *PELO* dependency. (**a**) Pearson correlation of relative copy number for genes on cytoband 9p21.3 with *PELO* dependency scores in 9p21.3^−/−^ cell lines. Genes included in the Cas12a library targeting 9p21.3 in red. (**b**) Left figure: immunoblot of FOCAD, PELO, Vinculin levels in KP4. Right figure: relative viability with indicated DOX-inducible gRNAs treated with DMSO or DOX. (**c**) Frequency of select alterations in TCGA dataset (*n* = 10,715 profiled tumors for HOMDEL and *n* = 10,443 for MUT_DRIVER). (**d**) *TTC37* mRNA transcript expression versus number of *TTC37* microsatellite repeats in cell lines (*n* = 1407). (**e**) TTC37 protein levels versus number of *TTC37* microsatellite repeats in cell lines (*n* = 372). (**f**) *PELO* dependency versus *FOCAD* relative copy number in cell lines (*n* = 1100), with cell lines harboring *TTC37* indels (≤ 9 microsatellite repeats, *n* = 32) highlighted. (**g**) Left figure: immunoblot of TTC37, PELO and β-actin. Right figure: relative viability of KM12 stably expressing indicated cDNA ± DOX-induced *PELO* KD. (**h**) Immunoblot of TTC37, PELO and β-actin. Relative viability of DLD1 stably expressing indicated cDNA ± DOX-induced *PELO* KD. (**i**) *PELO, KRAS, BRAF* dependency in cell lines with or without their corresponding biomarker and in 4 immortalized non-cancerous cell lines. Data are mean ± s.e.m. of biological replicates: *n* = 3 in (**b**); *n* = 3 except for *TTC37* cDNA DOX- where *n* = 2 in (**g**) and *n* = 3 in (**h**). For box plots in (**i**), the center line marks the median, the edges of the box represent the 25th–75th percentiles, and the whiskers span minimum-maximum values. Significance was calculated as follows: (**b**) left-tailed Student’s t-test, (**g,h**) right-tailed Student’s t-test, (**i**) left-tailed Student’s t-test. Representative data from one experiment are shown. All experiments were performed twice. For immunoblots, Vinculin and β-actin were used as loading controls. Source Data

We next pursued the lesion in 9p21.3^−/−^ cancers that confers *PELO* dependency. On the basis of the hypothesis that loss of a gene on 9p21.3 confers *PELO* dependency, we analysed DepMap for 9p21.3 genes that were correlated with *PELO* dependency in 9p21.3^−/−^cell lines. These data identified several 9p21.3 genes for which loss was similarly and strongly associated with *PELO* dependency, probably because these genes are frequently codeleted (Extended Data Fig. 3a). We thus performed a loss-of-function CRISPR–enhanced *Acidaminococcus* sp. Cas12a variant (enAsCas12a) screen with a focused library targeting eight 9p21.3 genes (Extended Data Fig. 3a) in combination with CRISPRi-mediated *PELO* knockdown^35^. We used KP4 (9p21.3 short deletion/MSS) cells, in which we saw no significant impairment in viability on *PELO* depletion (Fig. 2a). We transduced the enAsCas12a 9p21.3 gRNA library in KP4 cells expressing *dCas9*–*KRAB* and *PELO *gRNA 1 and 2, or Ch2-2 control (Fig. 3a). We observed that the mRNA surveillance gene *FOCAD* was the only gene whose knockout led to preferential impairment in cells with *PELO* depletion compared with control cells (left-tailed Wilcoxon rank-sum test, effect size = −0.786, *n* = 552, *P* = 1.80 × 10^−19^; Fig. 3a).

To evaluate whether *FOCAD* loss is necessary and/or sufficient for *PELO* dependency, we engineered three cell lines with or without *FOCAD*. We first interrogated whether *FOCAD* deletion was sufficient to confer dependency on *PELO* for viability. To do this, we knocked out *FOCAD* in two cell lines, WM793 (9p21.3^+^/MSS) and KP4 (9p21.3 short deletion/MSS) (Fig. 3b and Extended Data Fig. 3b). In tandem, a DOX-inducible CRISPRi gRNA targeting *PELO* was introduced into cells. We found that *PELO* was required for viability in these cell lines only upon *FOCAD* deletion (left-tailed Student’s *t*-test, WM793: effect size = −0.636, *n* = 6, *P* = 1.99 × 10^−7^; KP4: effect size = −0.51, *n* = 9, *P* = 8.79 × 10^−6^; Fig. 3b and Extended Data Fig. 3b), demonstrating that *FOCAD* deletion is sufficient to induce a dependency on *PELO*. We next investigated whether *FOCAD* loss was necessary for *PELO* dependency. For this purpose, we used MIA PaCa-2, a 9p21.3^−/−^/MSS model with biallelic loss of *FOCAD*. We exogenously expressed *FOCAD, PELO*, or control luciferase (*Luc*) cDNA and suppressed *PELO* expression with DOX-inducible *PELO *gRNA 2. In the presence of the control *Luc* cDNA, MIA PaCa-2 cells remained highly dependent on *PELO* for survival. By contrast, expression of *FOCAD* cDNA rescued MIA PaCa-2 viability (right-tailed Student’s *t*-test, effect size = 0.470, *n* = 6, *P* = 0.007; Fig. 3c) to a similar degree to *PELO* cDNA (right-tailed Student’s *t*-test, effect size = 0.479, *n* = 6, *P* = 0.005). Thus, our data show that *FOCAD* deletion is both necessary and sufficient for *PELO* dependency in the 9p21.3^−/−^ context.

Having refined our predictive biomarker for *PELO* dependency to *FOCAD* in 9p21.3^−/−^ cancers, we next queried the prevalence of *FOCAD* copy number in primary tumour samples. Using The Cancer Genome Atlas (TCGA) Pan-Cancer dataset, we found that *FOCAD* was deeply deleted in 335 out of 10,715 (3.1%) samples with consensus homozygous deletion calls^36^ (Extended Data Fig. 3c). In this context, biallelic *FOCAD* deletion is a relatively frequent cancer alteration, occurring more frequently than putative driver *EGFR* mutations (1.8%, 192 out of 10,443 samples; Extended Data Fig. 3c).

We next sought to determine the mechanistic basis for *PELO* dependency in the MSI-H context. We proposed the hypothesis that MSI-H-associated mutations in one or more genes sensitize cells to PELO depletion. Hence, we correlated *PELO* dependency with insertion–deletion mutations common in MSI-H cancers by inferring repeat length per microsatellite with MSIsensor2 for each cell line^28^ (Fig. 3d). *PELO* dependency was most strongly correlated with mutations in a microsatellite from chr. 5: 95507014–95507025 (Pearson’s *R* = 0.656, *n* = 73, *q* = 8.49 × 10^−7^), a sequence of 11 thymidines in the splicing acceptor site at intron 29 of the SKIc member *TTC37* (also known as *SKIC*3). We observed that deletions of thymidines in this microsatellite corresponded with increased *PELO* dependency in MSI-H cell lines (Fig. 3e). We also noted that *TTC37* expression scored as a potential biomarker in our univariate analysis that identified MSI-H score (Fig. 1e), so we reasoned that deletions at this site might alter splicing and destabilize *TTC37* mRNA. We found that nearly all cell lines with two or more thymidine deletions in the *TTC37* intron 29 polythymidine track were MSI-H, expressed lower levels of the dominant protein-coding transcript (ENST00000358746) and higher levels of an alternative non-coding transcript (ENST00000508181), and possessed reduced TTC37 protein levels^37^ (Extended Data Fig. 3d,e). These data indicate that *TTC37* intron 29 polythymidine deletions might be responsible for the reduced TTC37 protein levels, a finding that will need to be confirmed with future functional studies. We also observed that *TTC37* mutations more specifically predicted *PELO* dependency in *FOCAD*-intact cell lines than MSI-H status alone (Fig. 1d and Extended Data Fig. 3f).

To evaluate the role of *TTC37* loss in *PELO* synthetic lethality, we studied whether *TTC37* loss was sufficient and/or necessary for *PELO* dependency in MSI-H cells. First, we knocked out *TTC37* with CRISPR–enAsCas12 in KP4 cells (9p21.3 short deletion/MSS). We then treated cells with DOX to induce a CRISPRi gRNA targeting *PELO*. We observed that *PELO* knockdown impaired the viability of *TTC37*-deleted but not control KP4 cells (left-tailed Student’s *t*-test, effect size = −0.433, *n* = 9, *P* = 4.55 × 10^−4^; Fig. 3f). These data demonstrate that *TTC37* loss is sufficient to confer a dependency on *PELO* for survival. We next asked whether *TTC37* loss was necessary for *PELO* dependency in the MSI-H context. We exogenously expressed *TTC37* or *Luc* cDNA in three MSI-H colorectal cancer cell lines, HCT116, KM12 and DLD1, all containing a DOX-inducible *PELO* knockdown system. We found that exogenous expression of *TTC37* but not *Luc* rescued the viability of all three MSI-H cell lines from *PELO* knockdown (right-tailed Student’s *t*-test; HCT116: effect size = 0.505, *n* = 6, *P* = 3.2 × 10^−5^; KM12: effect size = 0.492, *n* = 6, *P* = 5.70 × 10^−7^; DLD1: effect size = 0.407, *n* = 6, *P* = 9.98 × 10^−7^; Fig. 3g and Extended Data Fig. 3g,h). Together, these data demonstrate that functional *TTC37* impairment is sufficient and necessary for *PELO* dependency in the MSI-H setting and could serve as a refined predictive biomarker for *PELO* dependency.

Next, we compared the relationships between our proposed biomarkers and *PELO* dependency with well-characterized dependency–biomarker relationships. These analyses showed the differential *PELO* dependency between biomarker-positive (*FOCAD* deletion or *TTC37* mutations) and biomarker-negative cell lines to be comparable with the relationships between *KRAS* dependency and *KRAS* mutations or *BRAF* dependency and *BRAF* mutations. Similarly, *PELO* biomarker-positive cell lines were significantly more dependent on *PELO* than non-cancerous immortalized cell lines (left-tailed Student’s *t*-test, effect size = −0.981, *n* = 75 PELO biomarker-positive cell lines, *n* = 4 non-cancerous cell lines, *P* = 0.001; Extended Data Fig. 3i).

**Fig. 4:**
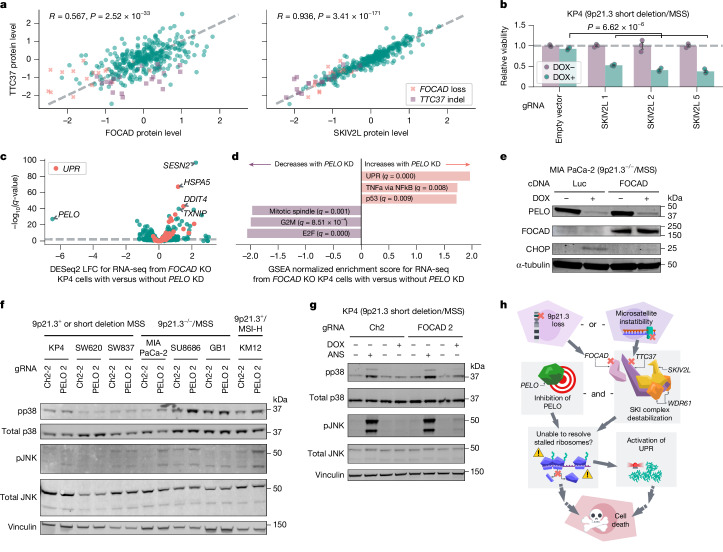
Evaluating loss of the SKIc as the common thread of *PELO* dependency. **a**, Comparison of TTC37, FOCAD and SKIV2L protein levels across cell lines (*n* = 375) with *FOCAD* loss (relative copy number < 0.4, *n* = 17) or *TTC37* insertion–deletion (indel; ≤9 microsatellite repeats, *n* = 23) indicated. **b**, Relative viability of KP4 cells with the indicated CRISPR knockout gRNAs ± DOX-induced *PELO* knockdown. **c**, *q*-values from DESeq2 Wald test for differential expression between RNA sequencing from KP4 *FOCAD* knockout cells ± DOX-induced *PELO* knockdown plotted against log_2_-transformed fold change. Genes in the Hallmark UPR gene set are highlighted. **d**, Hallmark gene set enrichment prerank results for log_2_-transformed fold changes shown in **c**. **e**, Immunoblots of PELO, FOCAD, CHOP and α-tubulin in MIA PaCa-2 cells with *Luc* or *FOCAD* cDNA ± DOX-induced *PELO* knockdown. **f**, Immunoblots of phospho-p38 (pp38, Thr180/Tyr182), total p38, phospho-JNK (pJNK, Thr183/Tyr185), total JNK and vinculin in the indicated cell lines with Ch2-2 gRNA or *PELO* knockdown. **g**, Immunoblots of pp38 (Thr180/Tyr182), total p38, pJNK (Thr183/Tyr185), total JNK and vinculin for KP4 cells with Ch2 gRNA or *FOCAD* knockout ± DOX-induced *PELO* knockdown ± anisomycin (ANS, positive control). **h**, Model of synthetic lethality between *PELO* dependency and 9p21.3^−/−^ and MSI-H cancers. Created in Lucid (lucid.co). Data are mean ± s.e.m. of biological replicates: *n* = 3 for (**b**–**d**). Significance was calculated using right-tailed Student’s *t*-test in **b**. Representative data from one experiment are shown. All experiments were performed twice, except for experiments in **c**,**d**,**f**, which were performed once. For immunoblots, α-tubulin and vinculin were used as loading controls. Source Data

**Extended Data Fig. 4:**
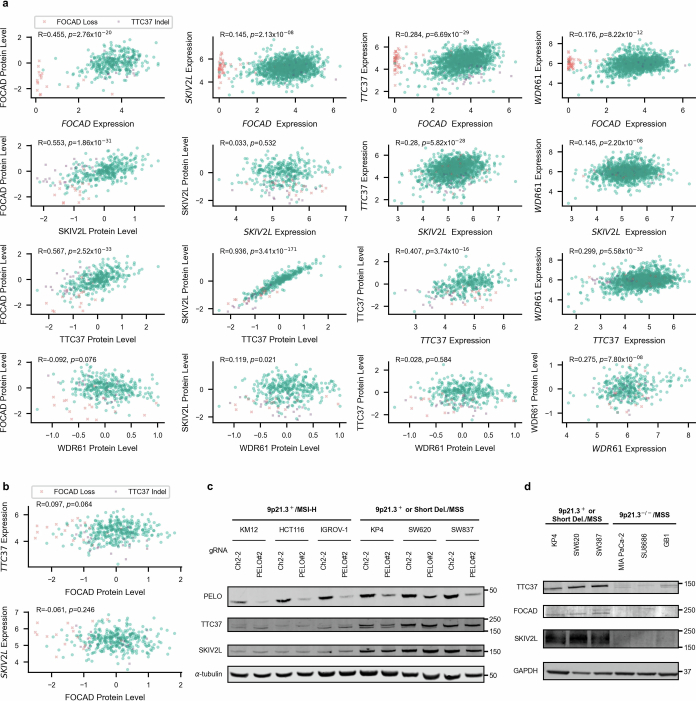
Evaluating the relationship between FOCAD and the SKIc subunits. (**a**) Comparison of SKIc protein levels (n = 375 cell lines) and mRNA expression levels (*n* = 1485) in cell lines. (**b**) Comparison of cell lines for FOCAD protein level and *SKIV2L* or *TTC37* mRNA expression levels (*n* = 369). (**c**) Immunoblot of PELO, TTC37, SKIV2L, α-tubulin in representative 9p21.3^+^/MSI-H and 9p21.3^+^ or Short Deletion/MSS cell lines. (**d**) Immunoblot of TTC37, FOCAD, SKIV2L, and GAPDH in representative 9p21.3^−/−^/MSS and 9p21.3^+^ or Short Deletion/MSS cell lines. Significance was calculated as follows: (**a, b**) Pearson’s R. Representative data from one experiment are shown. All experiments were performed once. For immunoblots, α-tubulin and GAPDH were used as loading controls. Source Data

**Extended Data Fig. 5:**
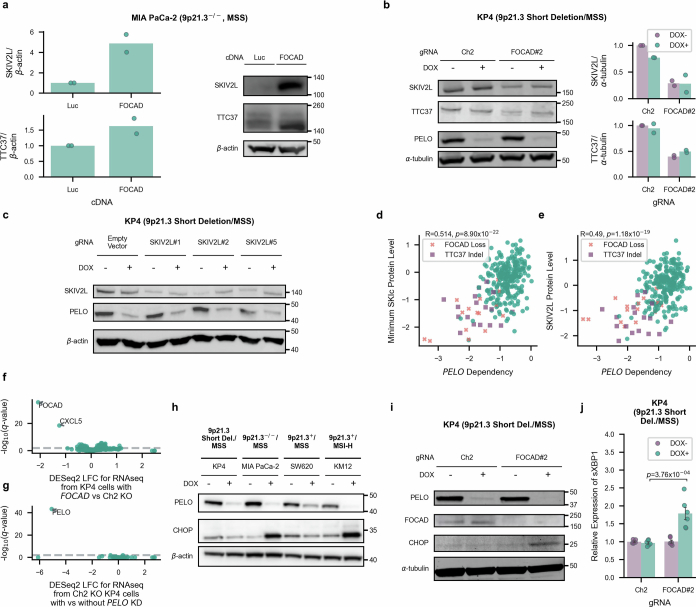
Evaluating the cellular consequences of PELO depletion in SKIc-deficient cancers. (**a**) Quantification (left) of immunoblot (right) for SKIV2L, TTC37, and β-actin protein levels for indicated cDNA in MIA PaCa-2. (**b**) Quantification (right) of immunoblot (left) for SKIV2L, TTC37, PELO, and α-tubulin protein levels for indicated enAasCas12a gRNAs ± DOX-induced *PELO* KD in KP4. (**c**) Immunoblot of SKIV2L, PELO, and β-actin in KP4 cells with indicated enAasCas12a gRNAs ± DOX-induced *PELO* KD. (**d**) Minimum SKIc protein level (calculated per cell line by taking the minimum of SKIV2L, TTC37, WDR61) versus *PELO* dependency across cell lines (*n* = 302). (**e**) SKIV2L protein level versus *PELO* dependency across cell lines (*n* = 302). (**f**) *q*-value from DESeq2 Wald test for differential expression between RNA-seq from KP4 control (Ch2 KO) and *FOCAD* KO cells plotted against log2-fold change. (**g**) *q*-value from DESeq2 Wald test for differential expression between RNA-seq from KP4 control (Ch2 gRNA) cells ± DOX-induced *PELO* KD plotted against log2-fold change. (**h**) Immunoblot of PELO, CHOP, and β-actin in indicated cell lines ± DOX-induced *PELO* KD. (**i**) Immunoblot of PELO, FOCAD, CHOP, and α-tubulin in KP4 cells with Ch2 gRNA or *FOCAD#2* ± DOX-induced *PELO* KD. (**j**) Reverse transcription quantitative polymerase chain reaction of spliced *XBP1* ± DOX-induced *PELO* KD in control (Ch2 gRNA) or *FOCAD*-deleted KP4 cells. Data are mean ± s.e.m. of biological replicates: *n* = 1 in two independent experiments in (**a,b**); *n* = 3 performed once in (**f, g**)**;** n = 1 in two independent experiments in (**h,i**). *n* = 3 for two independent experiments in (**j**). Significance was calculated as follows: (**d, e**) Pearson’s R, (**j**) right-tailed Student’s t-test. Representative data from two experiments are shown in (**a,b,j**). Data from one experiment are shown for (**c,f,g,h,i**). β-actin and α-tubulin were used as loading controls. Source Data

We next sought to investigate how *FOCAD* loss and *TTC37* mutations might converge to confer *PELO* dependency. TTC37 interacts with SKIV2L and WDR61 to form the SKIc, which promotes 3′→5′ exosome degradation of mRNA from stalled ribosomes^38^. Notably, FOCAD has been reported to physically interact with and stabilize the SKIc^39^. Thus, we proposed that protein levels of FOCAD, TTC37, SKIV2L and WDR61 would be correlated. Analyses of the Cancer Cell Line Encyclopedia (CCLE) proteomics dataset demonstrated positive correlations between FOCAD, TTC37 and SKIV2L protein levels^37^ (TTC37 and FOCAD: Pearson’s *R* = 0.567, *n* = 375, *P* = 2.52 × 10^−33^; TTC37 and SKIV2L: *R* = 0.936, *n* = 375, *P* = 3.41 × 10^−171^; SKIV2L and FOCAD: *R* = 0.553, *n* = 375, *P* = 1.86 × 10^−31^; Fig. 4a, Extended Data Fig. 4a). WDR61 protein levels showed little to no correlation with levels of the other three proteins (Extended Data Fig. 4a); this may reflect the dual role of WDR61 as a subunit of both the SKIc and the (PAF complex^37,40^. We examined *FOCAD*, *TTC37* and *SKIV2L* (also known as *SKIC2*) mRNA expression in DepMap and observed little correlation (Extended Data Fig. 4a). There was also no significant correlation between FOCAD protein levels and *TTC37* or *SKIV2L* mRNA expression, concordant with reports that FOCAD–SKIc interactions occur on the protein level^39,41^ (Extended Data Fig. 4b). Consistent with CCLE proteomic data, we observed decreased TTC37 and SKIV2L protein levels in 9p21.3^−/−^/MSS and 9p21.3^+^/MSI-H models with immunoblotting (Extended Data Fig. 4c,d). On the basis of these results, we asked whether FOCAD was required and/or sufficient to maintain TTC37 and SKIV2L protein stability. We exogenously expressed *FOCAD* cDNA in MIA PaCa-2 cells (9p21.3^−/−^/MSS) and observed increased TTC37 and SKIV2L protein levels (Extended Data Fig. 5a). In addition, we found that *FOCAD* knockout reduced SKIV2L and TTC37 protein levels in KP4 cells (9p21.3 short deletion/MSS) (Extended Data Fig. 5b). Together, these data support the claim that FOCAD is a crucial regulator of SKIV2L and TTC37 stability^39,41^.

These data suggest that both *FOCAD* deletions and *TTC37* mutations capture SKIc loss of function, thereby conferring increased reliance on *PELO* for survival. If functional loss of the SKIc was responsible for heightened *PELO* dependency, we proposed that *SKIV2L* deletion would confer increased *PELO* dependency. To investigate this, we knocked out *SKIV2L* with enAsCas12a gRNAs in KP4 cells (9p21.3 short deletion/MSS) and found depletion of SKIV2L with all three gRNAs (Extended Data Fig. 5c). In contrast to *SKIV2L*-intact KP4 cells, DOX-mediated *PELO* knockdown markedly impaired the viability of *SKIV2L* knockout KP4 cells (right-tailed Student’s *t*-test, effect size = 0.489, *n* = 12, *P* = 6.62 × 10^−6^; Fig. 4b). We also found that the minimum protein level of the three SKI complex members (TTC37, SKIV2L and WDR61) was highly predictive of *PELO* dependency (Pearson’s *R* = 0.514, *n* = 302, *P* = 8.90 × 10^−22^; Extended Data Fig. 5d). Similarly, *PELO* dependency could be predicted by SKIV2L protein levels alone (Pearson’s *R* = 0.49, *n* = 302, *P* = 1.18 × 10^−19^; Extended Data Fig. 5e), supporting our hypothesis that loss of SKIc function confers increased dependency on PELO for survival.

To assess how SKIc-deficient cancer cells responded to PELO suppression, we performed gene expression profiling. We observed minimal transcriptional responses following *FOCAD* knockout or *PELO* knockdown in parental KP4 cells (Extended Data Fig. 5f,g). By contrast, *PELO* knockdown resulted in a robust transcriptional response in *FOCAD*-deleted KP4 cells, with gene set enrichment analysis demonstrating upregulation of the unfolded protein response (UPR), a signalling network that responds to aggregated misfolded proteins^42–44^ (gene set enrichment analysis prerank, normalized enrichment score = 1.96, *q* = 0.000; Fig. 4c,d).

To validate these findings, we performed immunoblotting and found that *PELO* knockdown increased levels of UPR marker C/EBP homologous protein (CHOP) in 9p21.3^−/−^/MSS and 9p21.3^+^/MSI-H cells but not 9p21.3^+^/MSS cells (Extended Data Fig. 5h). We also found that restoring or destabilizing SKIc modulated UPR activation following *PELO* knockdown. *FOCAD* cDNA expression reduced CHOP levels in MIA PaCa-2 cells (9p21.3^−/−^/MSS) following *PELO* knockdown (Fig. 4e). In addition, CHOP levels and *XBP1* mRNA splicing, which are markers of UPR activation, increased upon *PELO* knockdown in *FOCAD*-deleted (but not *FOCAD*-intact) KP4 cells^45^ (right-tailed Student’s *t*-test, effect size = 0.821, *n* = 6, *P* = 3.76 × 10^−4^; Extended Data Fig. 5i,j). These data demonstrate that PELO depletion preferentially activates the UPR in the SKIc-deficient context. In addition to dissociating stalled ribosomes, the PELO–HBS1L complex has been shown to promote activation of ribosome-associated quality control, a process that degrades the nascent polypeptides that remain bound to 60S subunits after ribosomal splitting^46^. Thus, we propose a model in which loss of SKIc function to extract mRNA from stalled ribosomes uncovers an increased dependency on PELO–HBS1L to recruit ABCE1 and split the ribosome. In the SKIc-deficient context, loss of PELO–HBS1L activity to split ribosomes and activate ribosome-associated quality control may lead to accumulation of nascent polypeptides, activating the UPR.

UPR and other pathways responding to ribosomal stress, including the ribotoxic stress response, have been demonstrated to activate stress- and mitogen-activated proteins p38 and JNK^47–52^. Here, we evaluated whether *PELO* depletion activated the p38/JNK stress response in SKIc-deficient cells. We performed immunoblotting for phospho-p38 (Thr180/Tyr182) and phospho-JNK (Thr183/Tyr185), markers of p38 and JNK activation, respectively, across various cell lines representing 9p21.3^+^ or short deletion/MSS, 9p21.3^−/−^/MSS or 9p21.3^+^/MSI-H (Fig. 4f). We observed that *PELO* depletion preferentially induced both p38 and JNK phosphorylation in nearly all cell lines predicted to be SKIc deficient. In addition, *FOCAD* knockout in KP4 (9p21.3 short deletion/MSS) cells led to p38 and JNK phosphorylation following *PELO* depletion, mirroring the observed viability effects (Fig. 4g and Extended Data Fig. 3b). These data show that *PELO* loss and impairment of SKIc synergize to activate multiple stress pathways including the UPR and the p38/JNK stress response.

Our observations reveal that MSI-H associated mutations and large 9p21.3 deletions involving *TTC37* and *FOCAD*, respectively, independently impair the SKIc and confer a synthetic lethal relationship with *PELO* (Fig. 4h). As MSI-H and large 9p21.3 deletions are frequently observed in patients, a PELO-based therapeutic could have broad implications for clinical oncology. Although complete loss of PELO is embryonically lethal in mice, our data suggest that there is an exploitable therapeutic window with PELO inhibition^53^. Our data also support the development of predictive biomarkers based on SKIc protein detection, such as immunohistochemistry staining for SKIc subunits, to select patients who would be most likely to benefit from PELO-based therapeutics. Further studies will also be needed to explore the intersecting roles of PELO and the SKIc in mammalian cells. Notably, SKI2, the yeast homologue of SKIV2L has been functionally linked with Dom34, the yeast homologue of PELO, as both have been reported to help resolve stalled ribosomes^54^. Studies in *Saccharomyces cerevisiae* and *Caenorhabditis elegans* suggest that their PELO and SKIc homologues interact to resolve stalled ribosomes through recursive rounds of non-stop decay, particularly when a ribosome translates and arrests at the 3′ end of mRNA^55^. These models suggest that following endonuclease cleavage at the 5′ edge of the ribosome, the downstream stalled ribosome is rescued by PELO, while the SKIc clears the 3′ tail of the upstream mRNA fragment^54,56–58^. However, PELO and SKIc homologues are not synthetic lethal in *S. cerevisiae*, suggesting mechanistic differences in humans^59^. In *C. elegans*, the synthetic lethal relationship is nuanced and incomplete, with double SKIc and *PELO* homologue mutants exhibiting impaired fertility that is modulated by temperature^58^. Subsequent efforts will need to investigate why such a robust synthetic lethal phenotype arises in human cells, as well as the ribotoxic lesions that confer a requirement for PELO and/or SKIc and the downstream effectors that signal cell death. More broadly, our findings underscore the potential of DepMap to uncover novel therapeutic targets and functional interactions, opening new avenues for therapeutic interventions and fundamental biological understanding.

## Methods

### Genetic dependency and genomics data

We used CRISPR and genomics data from the DepMap 23Q4 release, including the CRISPRGeneEffect (gene dependency), OmicsCNSegmentsProfile (segment-level copy number), OmicsExpressionProteinCodingGenesTPMLogp1 (gene-level mRNA expression), OmicsExpressionTranscriptsTPMLogp1Profile (transcript-level mRNA expression) and Model (cell line metadata) files. The OmicsSignatures file from the DepMap 24Q2 release was also included. In addition, CCLE proteomics data were obtained from Nusinow et al.^37^. Alteration frequencies for FOCAD:HOMDEL, CDKN2A:HOMDEL, MTAP:HOMDEL, and EGFR:MUT_DRIVER in TCGA were obtained from cBioPortal’s TCGA Pan-Cancer Atlas 2018 (ref. ^60^). All genomic data were aligned to human genome assembly GRCh38. For more information, see ‘Data availability’ section.

### MSI scores

We used MSIsensor2 to score MSI from whole-genome sequencing or whole-exome sequencing samples without a matched normal^28^. The codebase for this algorithm can be found at https://github.com/niu-lab/msisensor2. For each hg38 BAM file, MSIsensor2 obtains a distribution of repeat lengths per microsatellite site from reads that are aligned with that region and saves them in an ‘output_dis’ file. The MSIsensor2 algorithm then uses the distribution of repeat lengths per site as input to a machine learning model that estimates the likelihood of a given site having lengthened or shortened as a result of MSI. The final MSIScore reported is the percentage of sites modified across the genome, with a recommended threshold of 20% for calling a sample MSI-H. The workflow description language (WDL) for running the MSIsensor2 workflow can be found in the DepMap Omics repo (https://github.com/broadinstitute/depmap_omics/blob/cf950f8c695cbf61db39d16718f999c622e1f16d/WGS_pipeline/msisensor2.wdl).

### 9p21.3^−/−^-associated dependency analysis

The genomic coordinates of 9p21.3 (chr. 9: 19900000–25600000) were identified as previously described^61^. A weighted average of the segment-level copy number calls was computed along this region, equivalent to the method used for producing gene-level copy numbers in DepMap. The relative copy number values for 9p21.3 were log_2_ transformed with a pseudocount of 1, log_2_(CN ratio + 1). We then identified 9p21.3^−/−^ and 9p21.3^+^ cell lines using each indicated relative copy number threshold between 0.2 and 0.6 (Fig. 1b and Extended Data Fig. 1a), and performed a left-tailed Student’s *t*-test between the two populations for all genetic dependencies in the CRISPR data (using scipy.stats.ttest_ind). All *q*-values were obtained using the Benjamini–Hochberg method (using scipy.stats.false_discovery_control).

### PELO biomarker analysis for 9p21.3^+^ cell lines

We aggregated expression, gene-level copy numbers and genomic signatures into a genomic feature matrix, excluding any with measurements in fewer than ten models. We then computed the Pearson correlation between *PELO* dependency in the 9p21.3^+^ models and genomic features in the feature matrix (using scipy.stats.pearsonr). All *q*-values were obtained using the Benjamini–Hochberg method (using scipy.stats.false_discovery_control).

### Association of PELO dependency with microsatellite repeat length

After running MSIsensor2, we obtained all output_dis files. We then took the weighted mean of each distribution in these files to find an approximate repeat length per microsatellite site and aggregated them to produce a matrix of microsatellite sites by cell lines. We then computed the Pearson correlation of *PELO* dependency in MSI-H models with the repeat lengths of all microsatellite sites (using scipy.stats.pearsonr). All *q*-values were obtained using the Benjamini–Hochberg method (using scipy.stats.false_discovery_control). The WDL for running the aggregate microsatellite repeats workflow can be found in the DepMap Omics repo (https://github.com/broadinstitute/depmap_omics/blob/cf950f8c695cbf61db39d16718f999c622e1f16d/WGS_pipeline/aggregate_microsatellite_repeats.wdl).

### RNA preparation and RNA sequencing analysis

KP4 cells with or without enAsCas12a-mediated *FOCAD* knockout were collected after 4 days of treatment with vehicle or DOX, and mRNA was isolated. After RNA integrity had been confirmed (RIN score > 9, High Sensitivity RNA ScreenTape) samples were submitted to ribosomal RNA depletion following the guidelines of the NEBNext kit. Libraries were prepared according to the NEBNext Ultra II Directional RNA Library Prep Kit for Illumina guidelines. Samples were sequenced using Illumina NextSeq2000.

Raw BCL files were demultiplexed using bcl2fastq (https://cumulus.readthedocs.io/en/latest/bcl2fastq.html); then, reads were aligned to hg38 using STAR (https://github.com/broadinstitute/depmap_omics/blob/a5308bc41227b86af47de545e14c38e7b8bf33f7/RNA_pipeline/star_wdl1-0.wdl). Next, RNA-SeQ2 and RSEM were run to obtain quality metrics and produce an expected counts matrix (https://github.com/broadinstitute/depmap_omics/blob/44518acd555e948df66178509ca1feb6c22c8b49/RNA_pipeline/RNA_stranded_rsem_rnaseqc2.wdl). The expected counts matrix was passed to DeseqDataset from pyDESeq2 v.0.4.9 (ref. ^62^), with design_factors referring to the knockdown + DOX condition and ref_level referring to the negative control (Chr2 knockdown + DOX− or *FOCAD* knockdown + DOX−) samples. Statistics were computed using DeseqStats to run a Wald test, after which lfc_shrink was applied to correct for low counts. For pathway enrichment, the resulting log_2_ fold changes were used as inputs to the prerank function from GSEApy v.1.1.3 to test gene sets in the Hallmark Collection^63^, as shown in Fig. 4d.

### Cell lines

All parental cell lines used in this study were obtained from the Broad Institute’s Cancer Dependency Map Project and CCLE bank, except for DLD1 cells, which were obtained from the laboratory of J. M. Schvartzman. All cancer cell lines were originally acquired from authorized cell line banks or academic collaborators; their specific sources can be found in Supplementary Table 1 or at DepMap Portal (https://depmap.org/portal/). Cancer cell lines were grown under their original culturing conditions according to supplier guidelines; these can be found in Supplementary Table 1. Short-tandem repeat profiling and mycoplasma testing (ABM, G238) were routinely performed in all cell lines.

### CRISPR constructs

For CRISPRi studies, gRNAs were inserted into all-in-one vector pXPR_023d vector expressing KRAB–dCas9–HA that contained the inactive *Cas9* (*dCas9*) fused to the *KRAB* transcriptional repression domain (Addgene, 202444)^64^. For DOX-inducible CRISPRi studies, the gRNAs were cloned to a pRDA_355 (Addgene,187159) vector, whereas the pLVd–Cas9–KRAB-MeCP2 vector was used for stable expression of chimeric protein dCas9–KRAB–MeCP2 (ref. ^34^). pLV–dCas9–KRAB–MeCP2 was a gift from X. Zhang^65^. For the CRISPR knockout enAsCas12a, vector pRDA_174neo was used to express enAsCasd12a. pRDA_174neo derived from pRDA174 (Addgene, 136476) by replacing the blasticidin-S deaminase gene with a neomycin resistance gene. The gRNAs were cloned into a pRDA_052Hygro, which was derived from pRDA_052 (Addgene, 136474) by replacing the puromycin resistance gene with a hygromycin resistance gene.

The gRNA sequences were designed using publicly available tool CRISPRick (https://portals.broadinstitute.org/gppx/crispick/public). The sequences for the individual gRNAs are shown in Supplementary Table 2. gRNA control Chr2-2 (Addgene, 125767) was previously described^31^.

### cDNA constructs

*FOCAD* (OHu16144), *PELO* (OHu17429) and *TTC37* (NM_014639.4) cDNAs were ordered from GenScript. pLX313_Firefly luciferase (Addgene, 118017) was used to Gibson assemble *FOCAD* and *PELO* cDNA in place of luciferase. We cloned Firefly luciferase and *TTC37* cDNA in a lentiviral expression vector containing an *EEF1A1* promoter to drive a puromycin resistance gene and cDNA expression, linked using a T2A peptide. Sanger sequencing of the vectors was performed to confirm sequence identity.

### Lentiviral production

Lentiviral production was performed using HEK293T cells following the guidelines described on the Broad Institute Genetic Perturbation Platform portal (http://portals.broadinstitue.org/gpp/public/). In brief, lentiviral particles were produced by transfecting HEK293T cells with packing (psPAX2; Addgene, 12260) and VSV-G envelope (pMD2.G; Addgene, 12259) plasmids using TransIT-LT1 transfection reagent (MIR 2306). Media were changed 8–16 h following transfection. Lentivirus-containing media were collected after 48 h.

### Viability assays

Viability was evaluated using CellTiter-Glo and/or clonogenic growth assay (Fig. 2a and Extended Data Fig. 2a,b). For experiments using a constitutive CRISPRi system, cell lines were transduced in a six-well plate and selected with puromycin for 3 days. We then confirmed that we had fully selected cells and reseeded cells on a 96-well plate for CellTiter-Glo or 24-well plates for clonogenic growth assays. The effects of *PELO* loss on viability were assessed between 10 and 14 days, depending on when the negative control reached confluency. Cells in 96-well plates were incubated with 50 µl of the CellTiter-Glo reagent for 20 min with constant shaking. Luminescence emission was measured using the PerkinElmer EnVision 2105.

For the clonogenic growth assay, cells were fixed with 4% paraformaldehyde at room temperature for 30 min. After removal of paraformaldehyde, cells were stained with 1% crystal violet solution with agitation at room temperature for 30 min. Cells were washed with water. Images of the stained cells were acquired, and dye was extracted with 10% acetic acid. Crystal violet absorbance values were read in triplicate at 595 nM with a PerkinElmer Envision 2105. Quantification values were normalized to either negative controls alone or negative controls and lethal controls, where negative controls were normalized to 1 and lethal controls to 0.

For DOX-inducible experiments, cells were first transduced with lentivirus harbouring inducible gRNAs and selected with the appropriate antibiotic. Cells were then transduced with lentivirus to stably express *dCas9*–*KRAB*–*MeCP2* and selected with blasticidin. Cells were then seeded on a 24-well plate. After 24 h, 0.5–1.0 μg ml^−1^ DOX was added to the cells and refreshed every 2–3 days. The viability effects were assessed between 8 and 14 days with the previously described clonogenic growth assay procedure.

Significance presented in figures was calculated as follows. In Fig. 2a and Extended Data Fig. 2a, left-tailed Student’s *t*-tests were performed comparing negative controls (Empty Vector and/or gRNA Ch2-2) with *PELO* gRNAs for biological replicates from the 9p21.3^+^ or short deletion/MSS, 9p21.3^−/−^/MSS or 9p21.3^+^/MSI-H groups to determine whether the relative viability upon *PELO* knockout was significantly lower than that of negative controls. In Fig. 2b, a right-tailed Student’s *t*-test was performed comparing differences between the means of *PELO* gRNAs and negative controls (empty vector, gRNA Ch2-2) per biological replicate for *Luc* and *PELO* cDNA overexpression to show that *PELO* overexpression after *PELO* knockdown led to a significant increase in viability. In Fig. 3b,f and Extended Data Fig. 3b, left-tailed Student’s *t*-tests were performed between the gRNA Ch2 negative control or empty vector and *FOCAD* or *TTC37* gRNA replicates in the DOX+ condition to show loss of viability upon biomarker knockout alongside *PELO* knockdown. In Fig. 3c,g and Extended Data Fig. 3g,h, right-tailed Student’s *t*-tests were performed between the *Luc* negative control and *FOCAD* or *TTC37* cDNA replicates in the DOX+ condition to show the increase in viability on biomarker overexpression in the presence of *PELO* knockdown. In Fig. 4b, a right-tailed Student’s *t*-test was performed to show that the differences between DOX+ and control replicates was significantly larger in the *SKIV2L* gRNA replicates compared with the empty vector replicates, indicating that *SKIV2L* knockout alongside *PELO* knockdown lead to a decrease in cell viability. All Student’s *t*-tests were performed with scipy.stats.ttest_ind.

### Immunoblot analysis

For experiments using anisomycin (A9789) as a positive control for ribosome stalling, cells were treated with 2 μg µl^−1^ of anisomycin (diluted in dimethyl sulfoxide) for 20 min^48^ (Fig. 4g). To prepare protein lysate, cells were washed with phosphate-buffered saline and either directly lysed with RIPA+ (RIPA buffer (ThermoScientific, 89901) with PhosSTOP phosphatase inhibitor (Sigma, 4906845001) and cOmplete protease inhibitor cocktail (Sigma, 11873580001)) and scraped into centrifuge tubes or trypsinized with TrypLE (Gibco, 12604013), quenched, washed, pelleted by centrifugation and lysed with RIPA+. Following 10 min incubation on ice, lysates were spun at 4 °C for 10–15 min at 20,000*g* to pellet cell debris. Supernatant was transferred to new tubes. Lysates were quantified using a Pierce BCA protein assay kit (Thermo Scientific, 23225) and normalized to 10–30 µg of total protein. Then, 10× NuPage sample reducing agent (Invitrogen, NP0009) and 4× NuPage LDS sample buffer (Invitrogen, NP0007) were added for final concentrations of 1× each. Lysates were heated and denatured at 70 °C for 10 min or 95 °C for 5 min. Samples were loaded and run on a 4–12% Bis–Tris gel (Invitrogen, NP0322BOX). For dry transfers, gels were transferred to a polyvinylidene fluoride membrane (Millipore, IPVH00010) using Invitrogen iBlot 2 (program P0). For wet transfers, gels were transferred with Bio-Rad Mini Trans-Blot Electrophoretic Transfer Cell (Bio-Rad, 1703930). Membranes were blocked with Intercept (PBS) blocking buffer (LI-COR Biosciences, 927-70001) for 30–60 min at room temperature. Membranes were incubated with primary antibodies overnight with 0.1% Tween-20 (Sigma, 11332465001). See Supplementary Table 3 for specific antibodies, dilutions used and other reagents. A near-infrared western blot detection system (LI-COR Biosciences) was used, following the manufacturer’s recommendations, with secondary antibodies goat anti-rabbit IRDye 800CW (LI-COR Biosciences, 926-32211; 1:5,000 or 1:20000), goat anti-rabbit IRDye 680LT (LI-COR Biosciences, 926-68021) and goat anti-mouse IRDye 800CW (LI-COR Biosciences, 926-32210). For detection of chemiluminescence signals, we incubated membranes with HRP-linked anti-rabbit IgG (Cell Signaling, 7074, 1:10,000; or Invitrogen, 31460, 1:20,000) secondary antibodies. Blots were developed with Cytiva ECL Prime Western Blotting Detection Agent (Cytiva, RPN2232) or SuperSignal West Atto Ultimate Sensitivity Chemiluminescent Substrate (Thermo Scientific, A38554), detected with a SynGene PXi or Amersham Imager TM 600 (29-0834-61), and processed with GeneSys image acquisition software (v.1.5.7.0) or ImageJ (v.1.53k).

### Quantitative PCR with reverse transcription

KP4 cells with or without *FOCAD* deletion were treated with vehicle or DOX for 4 days. After extraction and quantification, mRNA was reverse-transcribed into cDNA using a high-capacity cDNA reverse-transcription kit (Applied Biosystems, 4368813). We followed the supplier’s recommended protocol for SYBR-green master mix to perform quantitative PCR in a QuantStudio 7 Flex Real-Time PCR System (Applied Biosystems, 4484643). Relative expression was obtained after normalization with GAPDH. Primer sequences were as follows. GAPDH forward: GGAGCGAGATCCCTCCAAAAT; GAPDH reverse: GGCTGTTGTCATACTTCTCATGG; XBP1 spliced forward: CTGAGTCCGAATCAGGTGCAG; XBP1 spliced reverse: ATCCATGGGGAGATGTTCTGG^45,66,67^.

### 9p21.3 CRISPR–enAsCas12a modifier screen

The enAsCas12a library (two gRNAs per vector, 200 gRNAs total) targeting 9p21.3 genes, pan-essential genes and negative controls was assembled into the vector pRDA_052Hygro with golden gate assembly. Following transformation and amplification in Stbl4 bacteria (Invitrogen, 11635018), plasmid DNA was purified (QIAGEN Plasmid Plus Maxi Kit, 12965). We confirmed assembly and gRNA representation as per the Broad Institute Genomic Perturbation Platform protocols (https://portals.broadinstitute.org/gpp/public/resources/protocols). We next generated lentivirus and determined the amount of viral supernatant to achieve a multiplicity of infection between 0.3 and 0.5.

To prepare for the screen, we introduced dCas9–KRAB–HA in place of Cas9 in pXPR_051 (Addgene, 125775) to create pXPR_051d_Ch2-2. We then replaced Ch2-2 gRNA with *PELO* gRNA 1 or 2. We transduced KP4 cells with pRDA_174neo to express enAsCas12a. We next transduced cells with pXPR_051d Ch2-2 gRNA, *PELO* gRNA 1 or 2. Cells were selected with 250 µg ml^−1^ of hygromycin (Thermo Fisher, 10687010). We confirmed initial and sustained knockdown of *PELO* in these cells by immunoblotting 5 days following transduction and sustained knockdown 28 days following transduction. Engineered KP4 cells were then transduced in triplicate with the enAsCas12a library and selected with 1 µg ml^−1^ puromycin (Invitrogen Thermo Fisher, A1113803). Cells were collected, and genomic DNA was isolated for each replicate at 5, 9 and 13 days following transduction. Genomic DNA was sequenced by the Broad Institute Genetic Perturbation Platform as per the Broad Institute Genomic Perturbation Platform protocols (https://portals.broadinstitute.org/gpp/public/resources/protocols). In brief, the gRNA library was PCR amplified and sequenced on an Illumina HiSeq 2500 in high-output mode. The sequencing read length was single-end 50 base pairs with an eight-base index barcode read. Data were analysed using the Broad Institute Picard Pipeline (https://broadinstitute.github.io/picard/).

Read counts were normalized and analysed with Chronos^68^ (https://github.com/broadinstitute/chronos). We set kernel_width = 3 and cell_efficacy_guide_quantile = 0.15 to account for the small library size; otherwise, default hyperparameters were used. The negative control Ch2-2 gRNA replicates were treated as one screen, and the *PELO* gRNA 1 and 2 replicates were treated as two separate *PELO* knockdown screens. The plasmid DNA of the library was used as the day 0 reference, and multitargeting gRNAs were removed. The gene effect scores were then scaled such that the median of negative controls was 0 and the median of positive controls was −1.

To confirm that *FOCAD* deletion caused the most significant difference between the control Ch2-2 gRNA and *PELO* knockdown arms, we focused on the late timepoint (day 21) read counts. First, we computed log fold change (LFC) using the plasmid DNA reference. Then, for each replicate, we determined the differences in LFC for the two *PELO* knockdown arms and the control Ch2-2 gRNA arm for all gRNAs. Finally, we ran a left-tailed Wilcoxon rank-sum test (scipy.stats.ranksums) comparing the differences for *FOCAD* and non-*FOCAD* gRNAs to determine the difference between the *PELO* arm LFC and control arm LFC.

### Patient-derived tumour organoids

All samples were obtained from patients with informed consent at the Dana-Farber Cancer Institute. All human research procedures were conducted under a protocol approved by the Dana-Farber Cancer Institute Institutional Review Board. Derivation of patient-derived organoids has been previously described for CCLF_CORE_001 (ref. ^31^) and for PANFR0127 and PANFR0071 (ref. ^69^). Models were propagated as previously described^69^. To transduce these models, we first dissociated cells from their extracellular matrix (Corning Matrigel Growth Factor Reduced, 354230) by digestion with TrypLE (Gibco, catalogue no. 12604021). Cells were then plated on six-well Ultra-Low Adherent Plates (Corning, 3471) in medium containing 8 µg ml^−1^ polybrene. Lentivirus was added, and plates were subjected to centrifugation at 1,000*g* for 1 h at 30 °C. The cells were placed in a 37 °C incubator. After 4–6 h of incubation, cells were transferred to an 15-ml conical tube and subjected to centrifugation at 300*g* for 5 min at 4 °C. The tube was placed on ice, and the cells were resuspended in Corning Matrigel extracellular matrix (Corning, 356231). Cells were then replated on tissue-culture-treated six-well plates (Corning, 3516) for propagation. Following transduction with the inducible gRNA vectors, this process was repeated to enable stable expression of *dCas9*–*KRAB*–*MeCP2*. We analysed mRNA sequencing data and observed that PANFR0127 lacked mRNA expression of *FOCAD*. PANFR0071 expressed *FOCAD* with a log*2*(transcript count per million) + 1 of 3.9.

For viability experiments, matrigel–tumour cell mixtures were seeded in duplicate or triplicate for each condition in a 24-well plate. One day following seeding, the growth medium was changed to contain 0.5–1.0 µg ml^−1^ of DOX or the same volume of dimethyl sulfoxide. Every 2–3 days, the medium was changed to refresh DOX. After 8–10 days, all medium was aspirated, and 500 µl of medium and 500 µl of CellTiter-Glo 3D (Promega, G9681) were added. After agitation for 30 min at room temperature, the mixture was transferred to a 96-well plate (Corning, 3903), and luminescence was measured with a PerkinElmer Envision 2105.

### Xenograft studies

Xenograft studies were performed in accordance with the Columbia University Institutional Animal Care and Use Committee (IACUC) animal protocol AC-AABT8654. IACUC guidelines on the ethical use and care of animals were followed. MIA PaCa-2 cells were engineered with stably expressed *dCas9*–*KRAB*–*MeCP2* (Addgene, 110821) and a DOX-inducible *PELO *gRNA 2 inserted into pRDA_355. Six-week-old female homozygous NU/J mice obtained from Jackson Laboratories (002019) were inoculated subcutaneously on their bilateral flanks with 7 × 10^6^ engineered MIA PaCa-2 cells. To confirm that our DOX-inducible CRISPRi system knocked down *PELO* in vivo, we engrafted four mice. When tumours reached approximately 150 mm^3^, mice were fed Teklad Global 18% Protein Rodent Diet containing 625 mg kg^−1^ DOX (Inotiv Teklad, TD.01306). Tumours were harvested at the indicated time points and homogenized by placing samples in Fisherbrand Pre-Filled Bead Mill Tubes (Fisher Scientific, 15-340-153) on a Fisherbrand Bead Mill 24 (Fisher Scientific, 15-340-163). The homogenizer was set to a speed of 6 m s^−1^ for one cycle of 30 s. Lysates were placed on ice for 2 min and then rotated at 4 °C for 30 min. The supernatant was transferred to 1.5-ml microfuge tubes and centrifuged at 13,000*g* for 15 min at 4 °C. After centrifugation, the supernatant was transferred to fresh microfuge tubes and centrifuged again under the same conditions. The supernatant was again transferred to fresh microfuge tubes and passed through a 30-g insulin syringe ten times (EasyTouch NDC: 08496-3055-01). Before fractionating, the lysates were treated with 0.35 µl of benzonase (Sigma-Aldrich, 71206) for 1 h on ice. Immunoblots were repeated three times. Extended Data Fig. 2c shows representative results from one immunoblot.

For growth measurements, a total of 11 mice were subcutaneously inoculated on their bilateral flanks with MIA PaCa-2 cells. Tumours were measured twice weekly (for the first 66 days) or weekly (after 66 days) with callipers, and the tumour volumes were calculated using the formula 1/2 × (width^2^ × length). When primary tumours reached approximately 150 mm^3^ (range 50–300 mm^3^), mice were randomized to DOX feed or to remain on standard feed. In three of the 11 mice (two randomized to standard feed, one randomized to DOX), one tumour reached the size criteria for randomization, but the contralateral tumour remained under 50 mm^3^. In this scenario, we omitted the smaller tumour from our study. In total, we had ten tumours in six mice in our standard feed arm and nine tumours in five mice for our DOX feed arm. Mice remained on their respective diets until day 66, at which point all mice on the regular diet were euthanized owing to tumour size end points. At this point, all mice were transitioned back to a regular diet. No experiment exceeded the maximal tumour volumes set forth by the IACUC. This study was not blinded. No sample size calculations were performed. The Kaplan–Meier plot in Fig. 2e was generated with the KaplanMeierFitter function from the Python package lifelines. The *P* value reported is from lifelines.statistics.pairwise_logrank_test.

### Material availability

Plasmids generated in this study have been deposited to Addgene (Supplementary Table 4).

### Reporting summary

Further information on research design is available in the Nature Portfolio Reporting Summary linked to this article.

## Online content

Any methods, additional references, Nature Portfolio reporting summaries, source data, extended data, supplementary information, acknowledgements, peer review information; details of author contributions and competing interests; and statements of data and code availability are available at 10.1038/s41586-024-08509-3.

## Supplementary information

Supplementary Information

Reporting Summary

## Source data

Source Data Fig. 1

Source Data Fig. 2

Source Data Fig. 3

Source Data Fig. 4

Source Data Extended Data Fig. 1

Source Data Extended Data Fig. 2

Source Data Extended Data Fig. 3

Source Data Extended Data Fig. 4

Source Data Extended Data Fig. 5

## Data Availability

DepMap 23Q4 data for Figs. 1b–f, 3d,e, 4a and Extended Data Figs. 1a,b, 3d–f,i, 4a,b, 5d,e are available via Figshare at https://plus.figshare.com/articles/dataset/DepMap_23Q4_Public/24667905/2.^24^ The DepMap 24Q2 OmicsSignatures file used in Figs. 1e–d, 3e and Extended Data Figs. 1b, 3d,e are available via Figshare at https://plus.figshare.com/articles/dataset/DepMap_24Q2_Public/25880521/1 (ref. ^70^). CCLE proteomics data shown in Fig. 4a and Extended Data Figs. 3d,e, 4a,b, 5d,e can be found in ref. ^37^. All other data for Figs. 1b–e, 3a,d,e, 4a,c,d and Extended Data Figs. 1a,b, 3a,d–f,i, 4a,b and 5d–g are available via Figshare at https://figshare.com/articles/journal_contribution/Borck_et_al_2025/27249741 (ref. ^71^). Source data are provided with this paper.

## Code availability

Code used for analysis can be found at via GitHub at https://github.com/broadinstitute/Borck-et-al-2025 (ref. ^72^).
