# Supplementary material for: SKI complex loss renders 9p21.3-deleted or MSI-H cancers dependent on PELO
Source: Nature. 2025 Feb 5;638(8052):1104–11. doi: 10.1038/s41586-024-08509-3 (PMC11864980; doi:10.1038/s41586-024-08509-3)
Supplement: Supplementary file 1 — Supplementary Information [file 41586_2024_8509_MOESM1_ESM.pdf]

nature portfolio

https://doi.org/10.1038/s41586-024-08509-3

Supplementary information

SKI complex loss renders 9p21.3-deleted or MSI-H cancers dependent on PELO

In the format provided by the authors and unedited

SKI complex loss renders 9p21.3-deleted or MSI-H cancers dependent on PELO

Patricia C Borck[1], Isabella Boyle[1], Kristina Jankovic[2], Nolan Bick[1], Kyla Foster[1], Anthony C Lau[1], Lucy I. Parker-Burns[2], Daniel A. Lubicki[1], Tianxia Li[2], Ashir A Borah[1], Nicholas J Lofaso[2], Sohani Das Sharma[2], Tessla Chan[2], Riya V. Kishen[2], Anisah Adeagbo[1], Srivatsan Raghavan[1,3], Elisa Aquilanti[1,4], John R Prensner[1,5], J Michael Krill-Burger[1], Todd R Golub[1,6], Catarina D Campbell[1], Joshua M Dempster[1], Edmond M Chan[1,2,7,8#], Francisca Vazquez[1#]

The following files can be found on Supplementary Information:

- **Supplementary Figure S1:** Western blot images
- **Supplementary Table S1:** Cell line information
- **Supplementary Table S2:** CRISPR guide sequence
- **Supplementary Table S3:** Antibody information
- **Supplementary Table S4:** Plasmids

The following files can be found on Figshare:

- **9p21_3_relative_copy_number.csv** - relative copy number for 9p21.3 cytoband
- **TTC37_transcript_expr.csv** - transcript expression for select TTC37 transcripts, taken from DepMap 23Q4 Public
- **microsatellite_repeats.csv** - repeat lengths extracted from MSISensor2
- **TCGA_alterations.csv** - alterations of certain genes in TCGA downloaded from cBioPortal
- **readcounts_9p21_3.csv** - raw readcounts from CRISPR modifier
- **guide_map_9p21_3.csv** - map of guides to genes in CRISPR modifier
- **sequence_map_9p21_3.csv** - map of sequences (replicates) to conditions (cell line names) in CRISPR modifier screen
- **RSEM_expected_count_RNAseq.csv** - RSEM expected counts for RNAseq
- **sample_metadata_RSEMseq.csv** - metadata for samples in RNAseq
- **hallmark_gene_sets_2023.csv** - Hallmark gene sets from mSigDB (h.all.v2023.2.Hs.symbols.gmt) reformatted to CSV
- **Ch2_KO_DOXpos_vs_neg_deseq2.csv** - DESeq2 results from KP4 with Ch2 KO for DOX+ vs DOX- condition
- **Ch2_KO_DOXpos_vs_neg_gsea_prerank.csv** - GSEA pre-rank results from KP4 with Ch2 KO for DOX+ vs DOX- condition
- **DOXneg_FOCAD_vs_Ch2_KO_deseq2.csv** - DESeq2 results from KP4 with Ch2 KO vs FOCAD KO for DOX- condition
- **DOXneg_FOCAD_vs_Ch2_KO_gsea_prerank.csv** - GSEA pre-rank results with Ch2 KO vs FOCAD KO for DOX- condition
- **FOCAD_KO_DOXpos_vs_neg_deseq2.csv** - DESeq2 results from KP4 with FOCAD KO for DOX+ vs DOX- condition

- **FOCAD_KO_DOXpos_vs_neg_gsea_prerank.csv** - GSEA pre-rank results from KP4 with FOCAD KO for DOX+ vs DOX- condition

Supplementary Figure 1

Used in Figure 2b upper panel

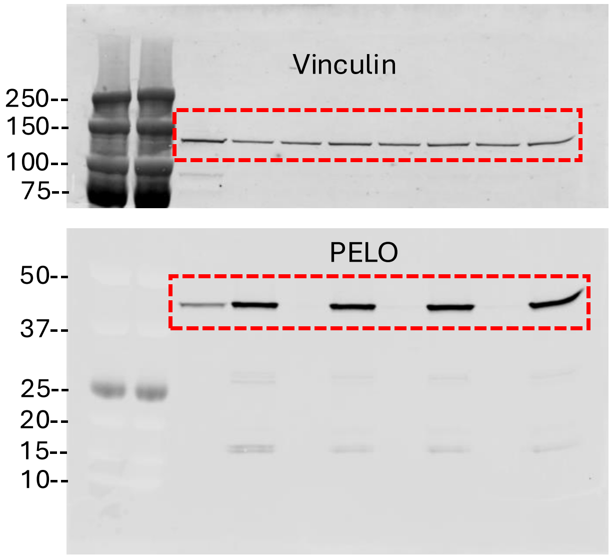

Used in Figure 2c left panel

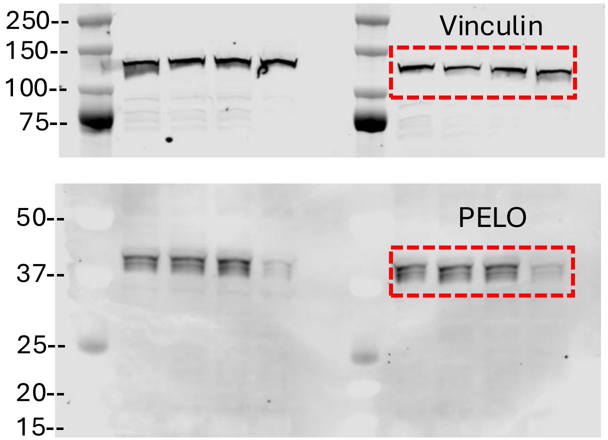

Used in Figure 2c middle panel

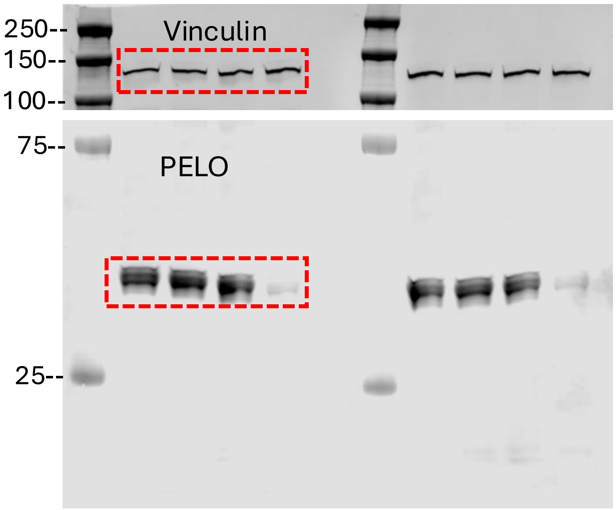

Used in Figure 2c right panel

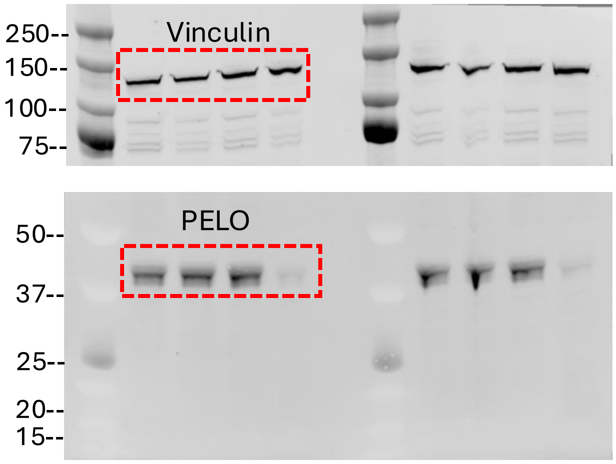

Supplementary Figure 1

Used in Ext Figure 2b upper panel

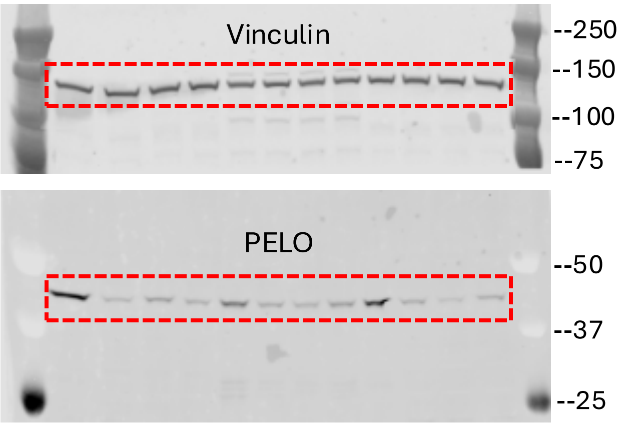

Used in Ext Figure 2b middle panel

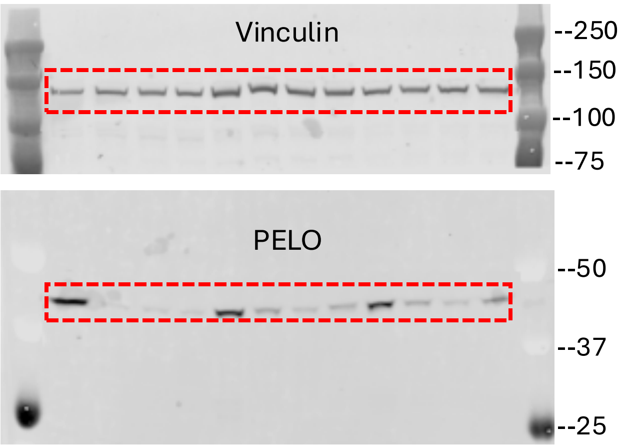

Used in Ext Figure 2b lower panel

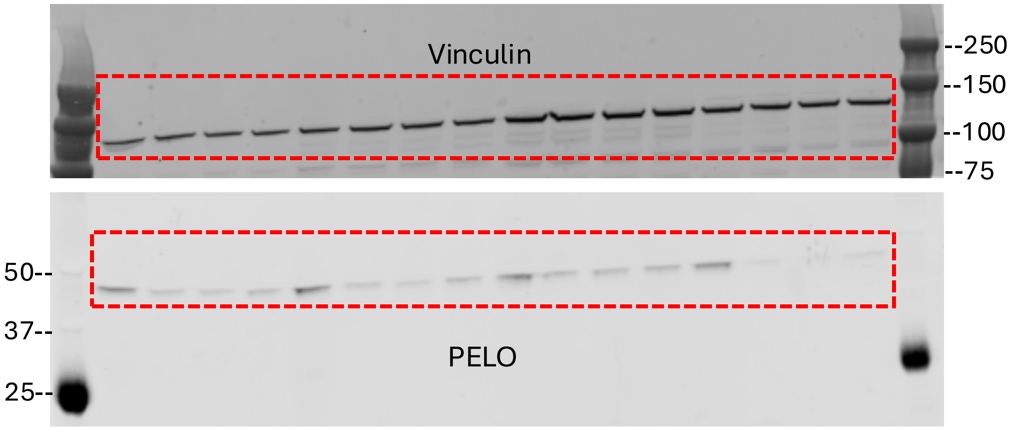

Used in Ext Figure 2c

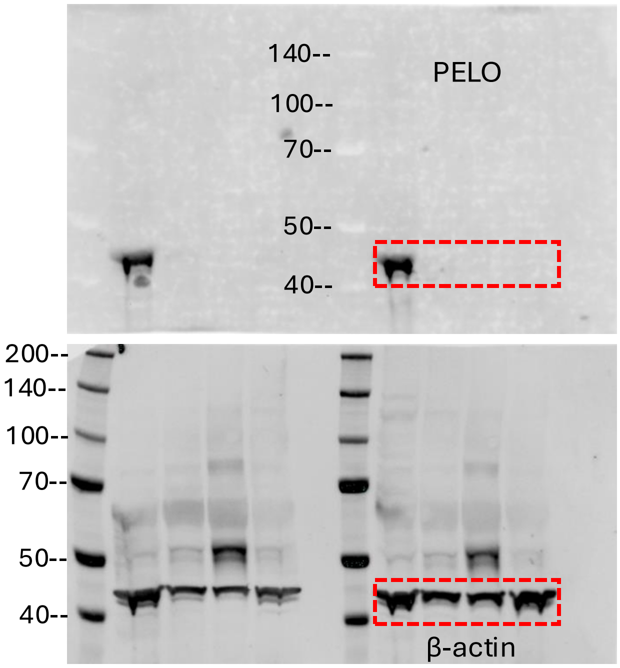

Supplementary Figure 1

Used in Figure 3b upper panel

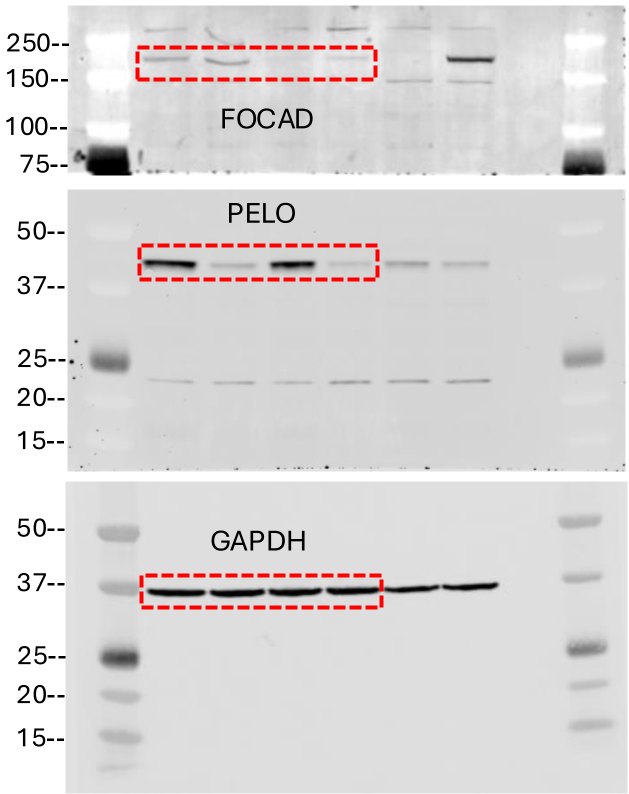

Used in Figure 3c upper panel

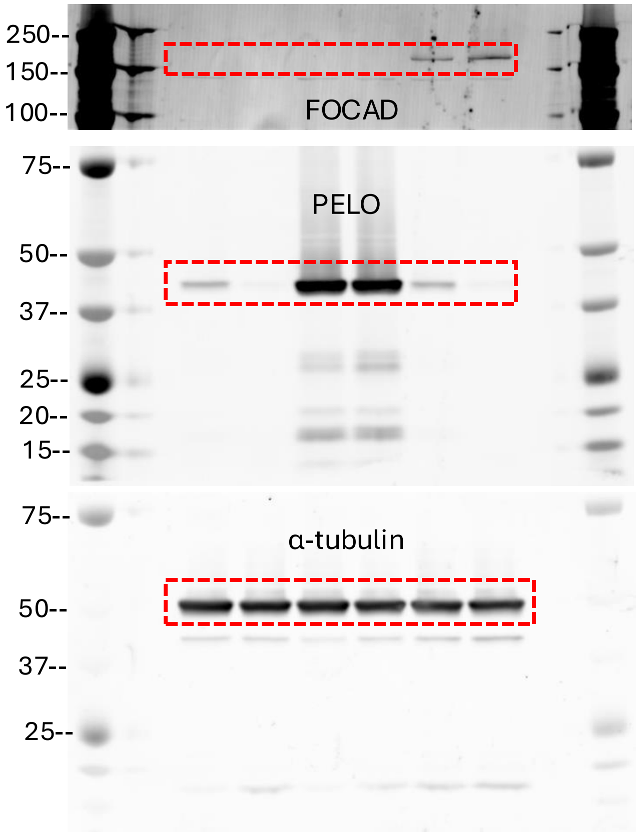

Used in Figure 3f upper panel

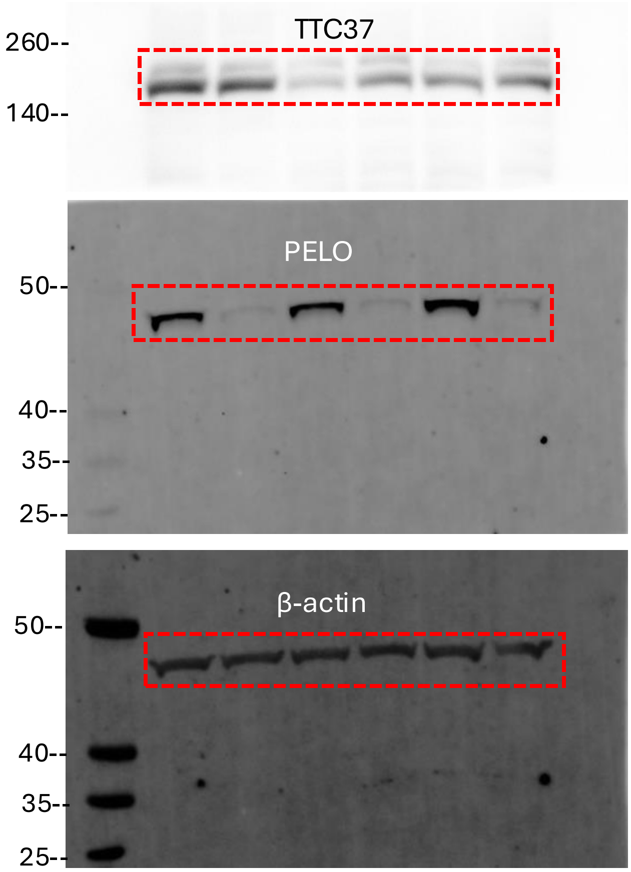

Used in Figure 3g upper panel

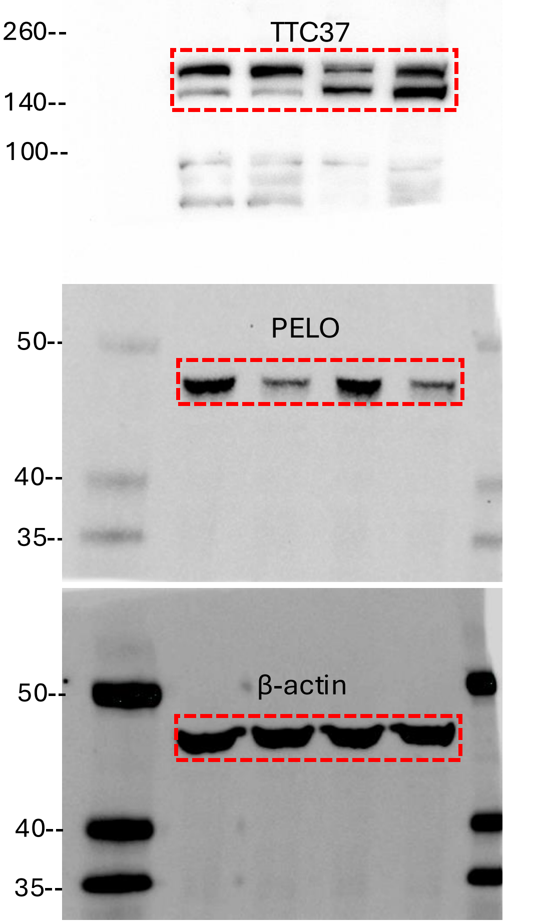

Supplementary Figure 1

Used in Ext Figure 3b

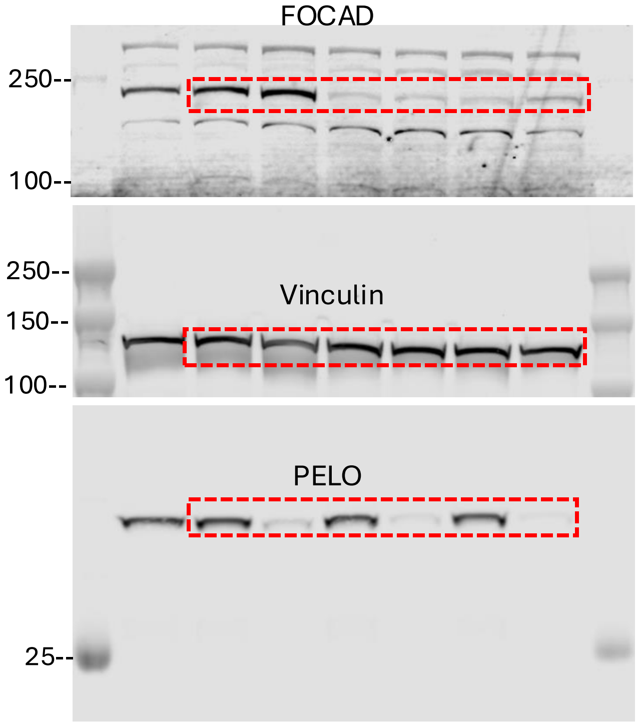

Used in Ext Figure 3g

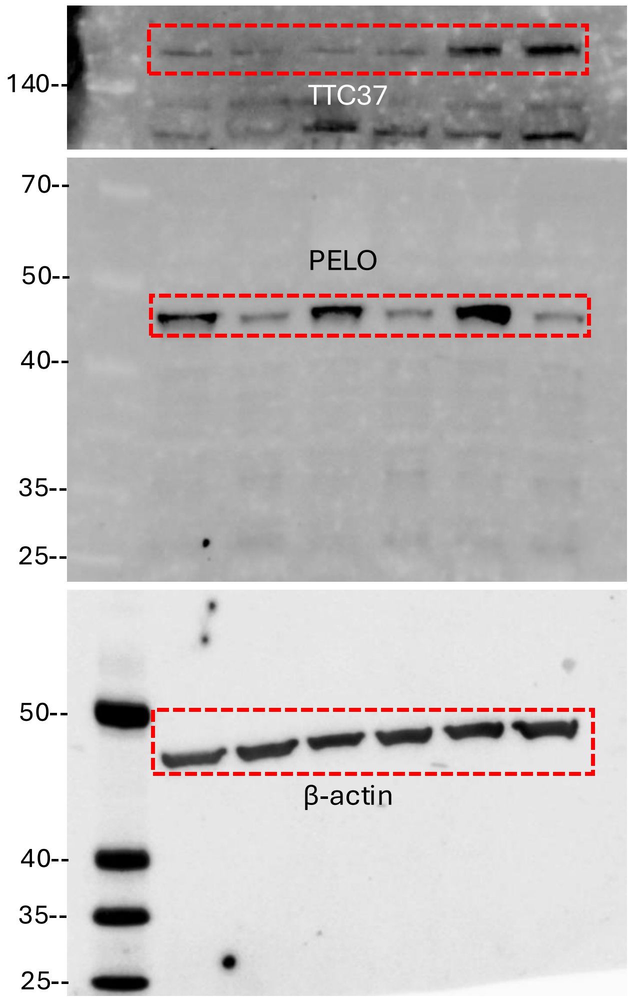

Used in Ext Figure 3h

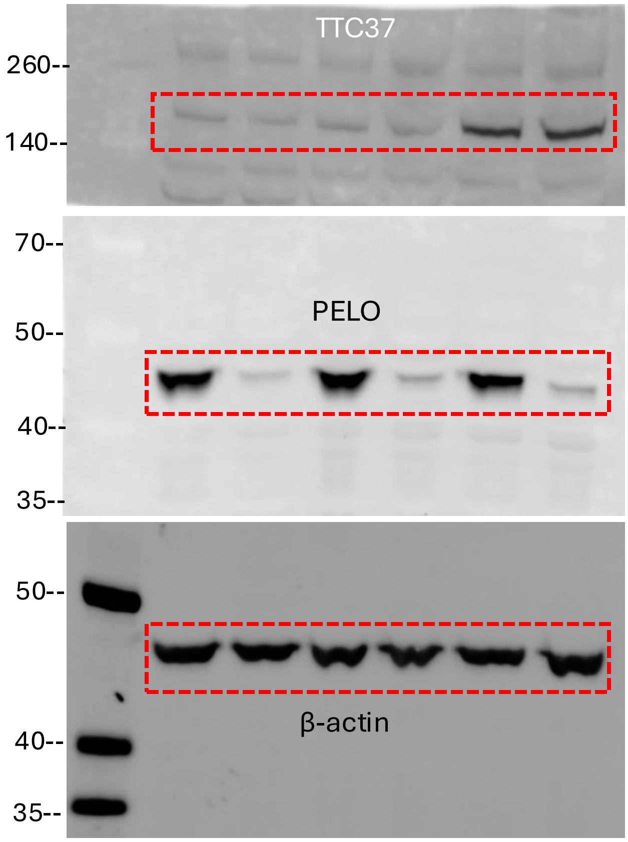

Supplementary Figure 1

Used in Figure 4e

Used in Ext Figure 5i

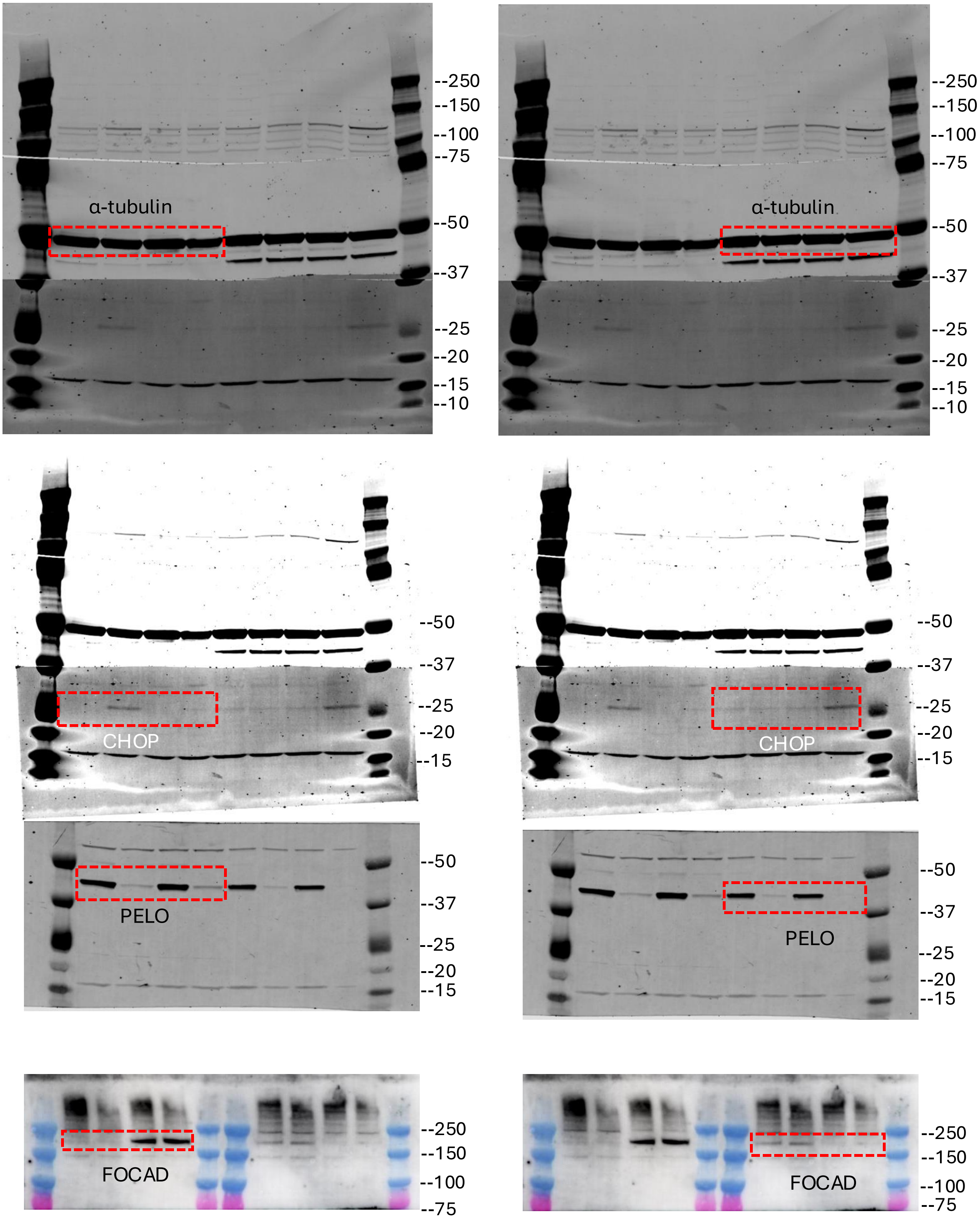

Supplementary Figure 1

Used in Figure 4f

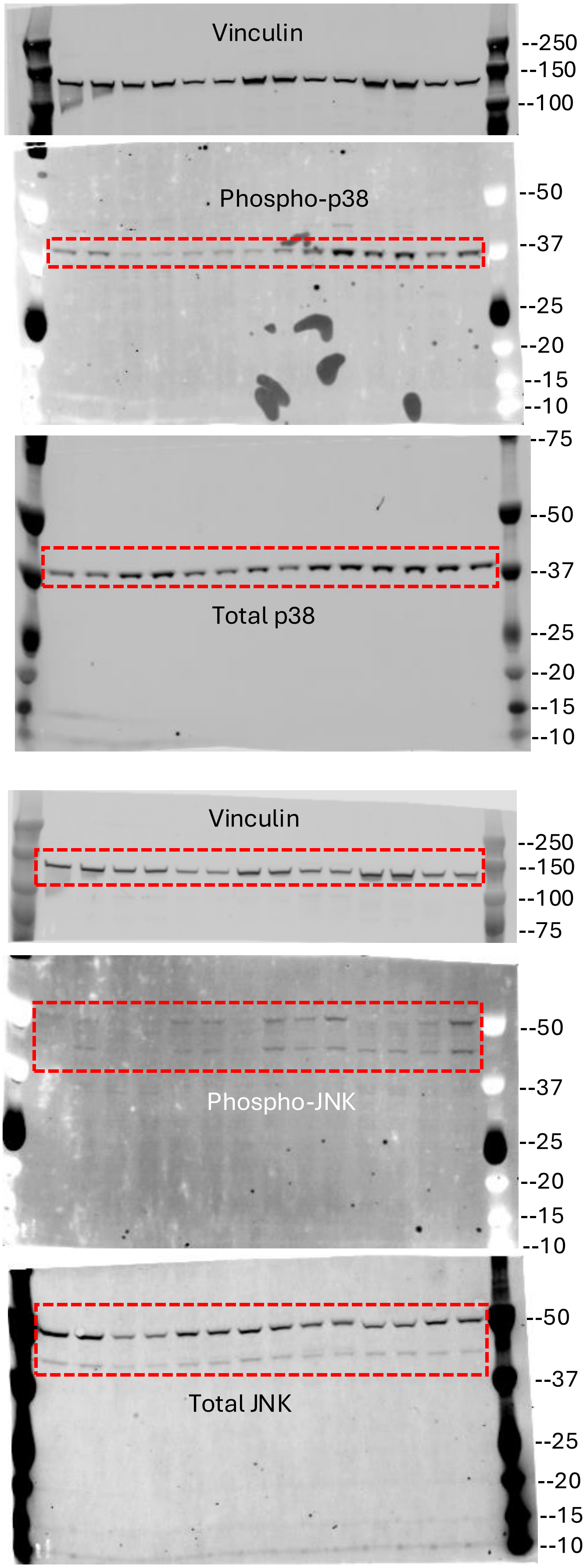

Used in Figure 4g

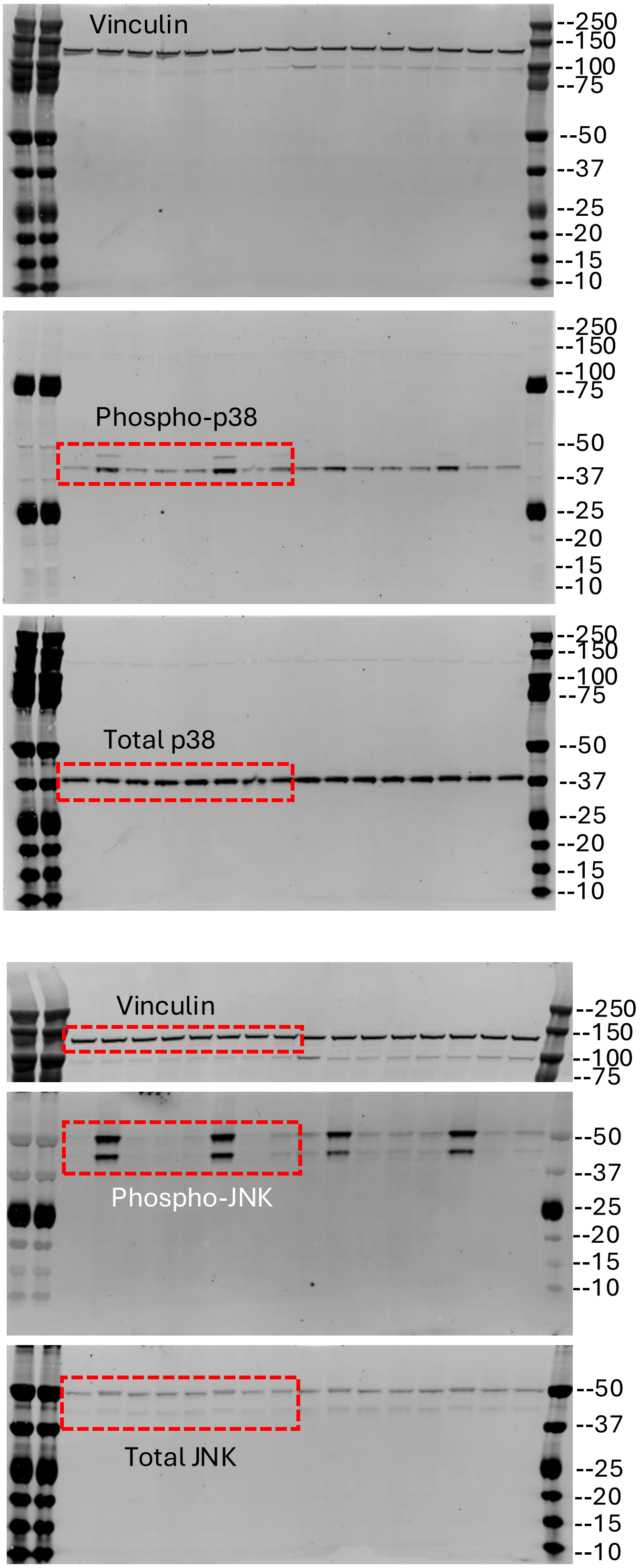

Supplementary Figure 1

Used in Ext Figure 4c

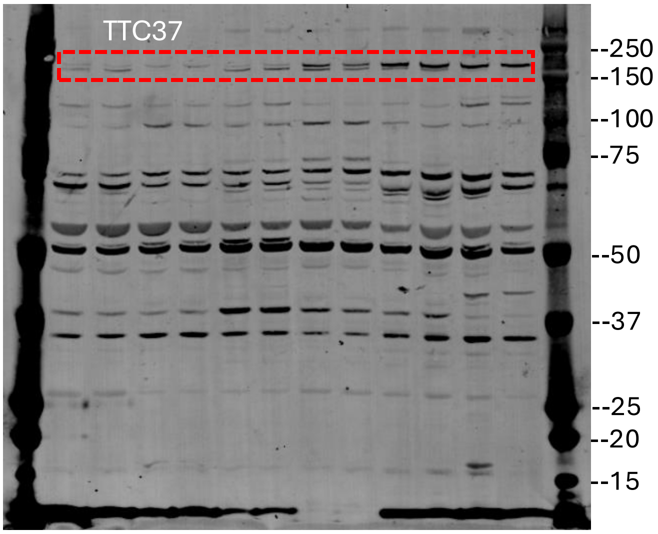

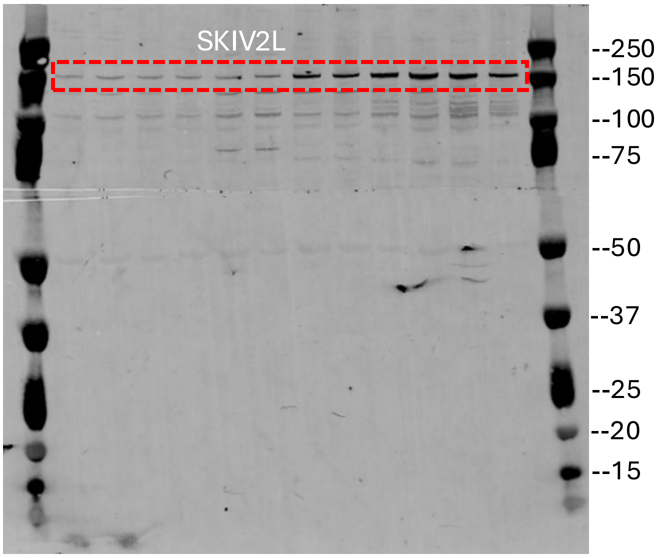

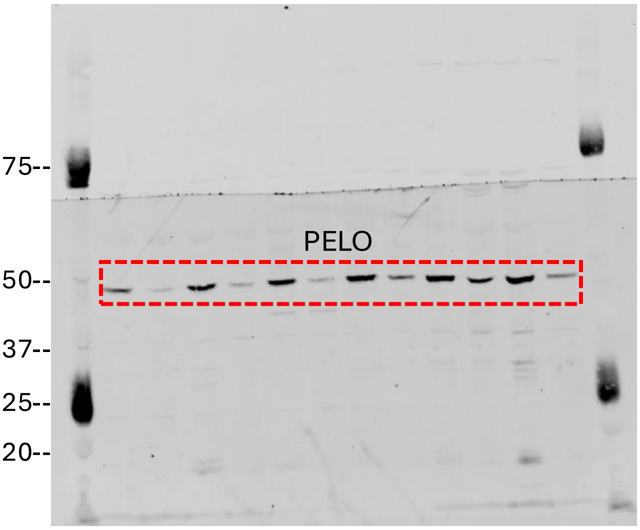

Used in Ext Figure 4c

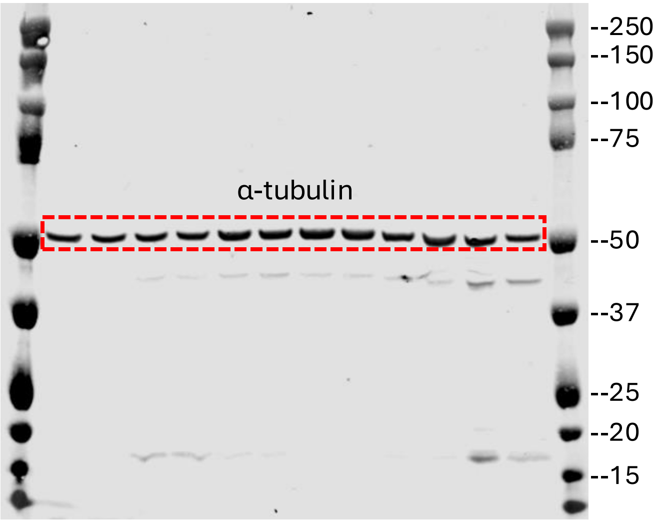

Used in Ext Figure 4d

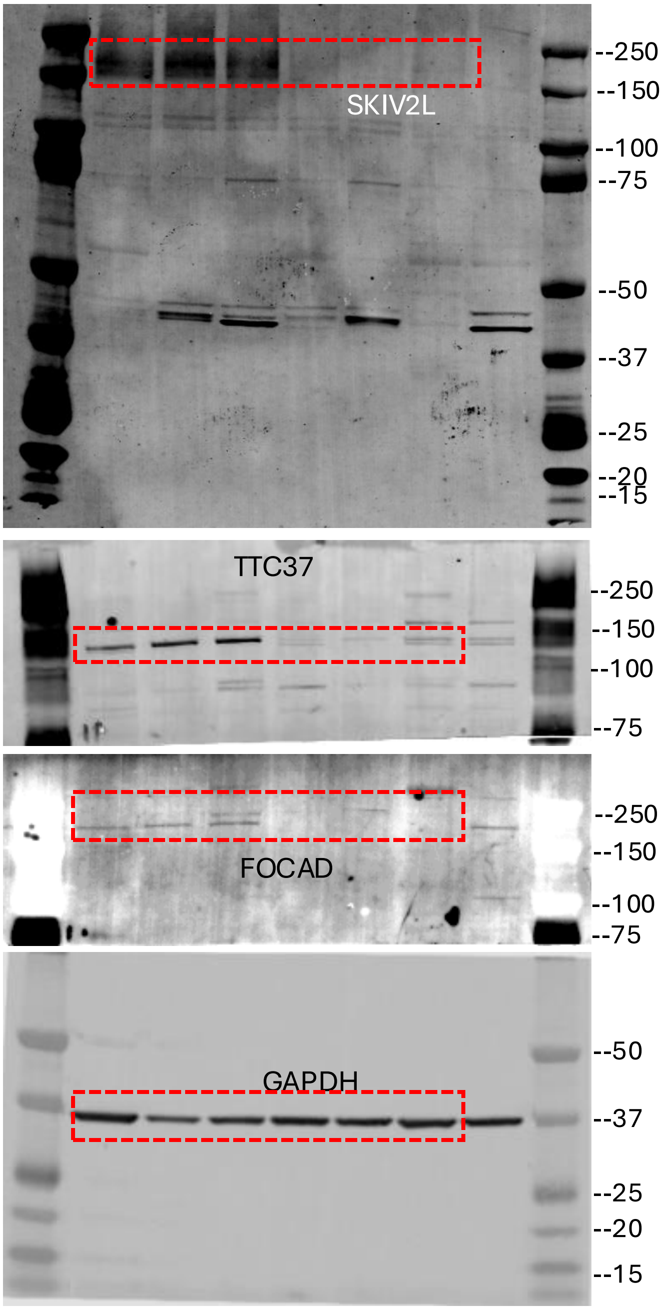

Supplementary Figure 1

Used in Ext Figure 5a

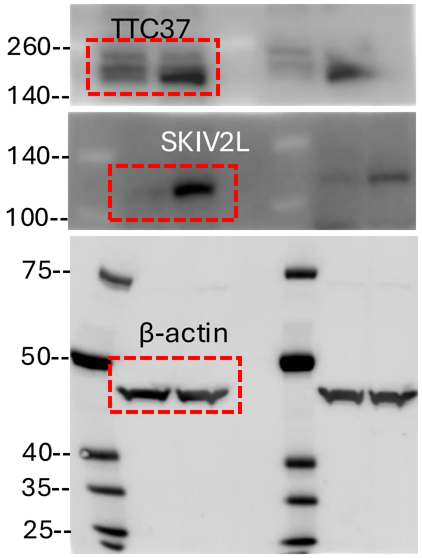

Used in Ext Figure 5b

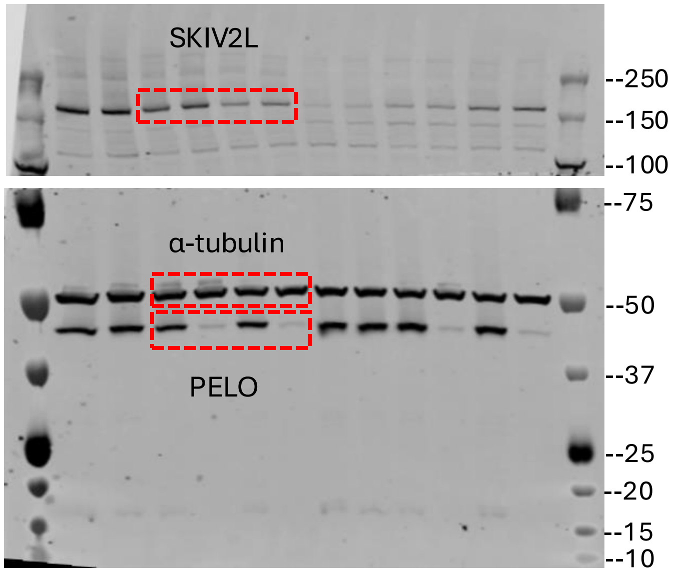

Used in Ext Figure 5b

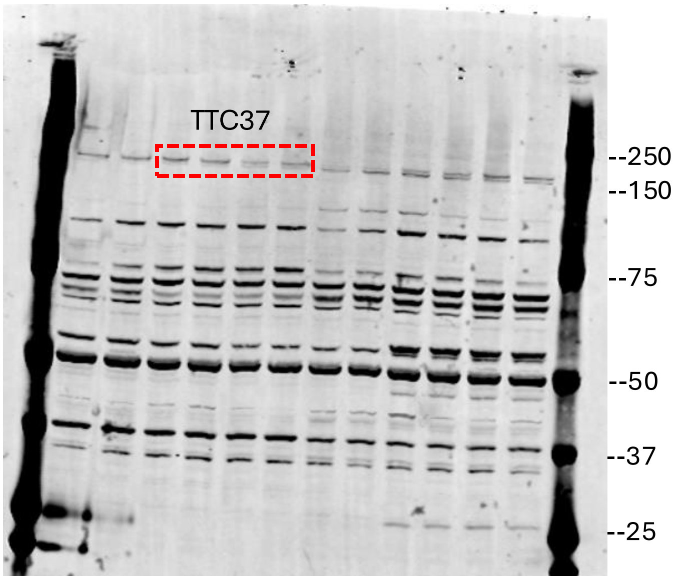

Used in Ext Figure 5c

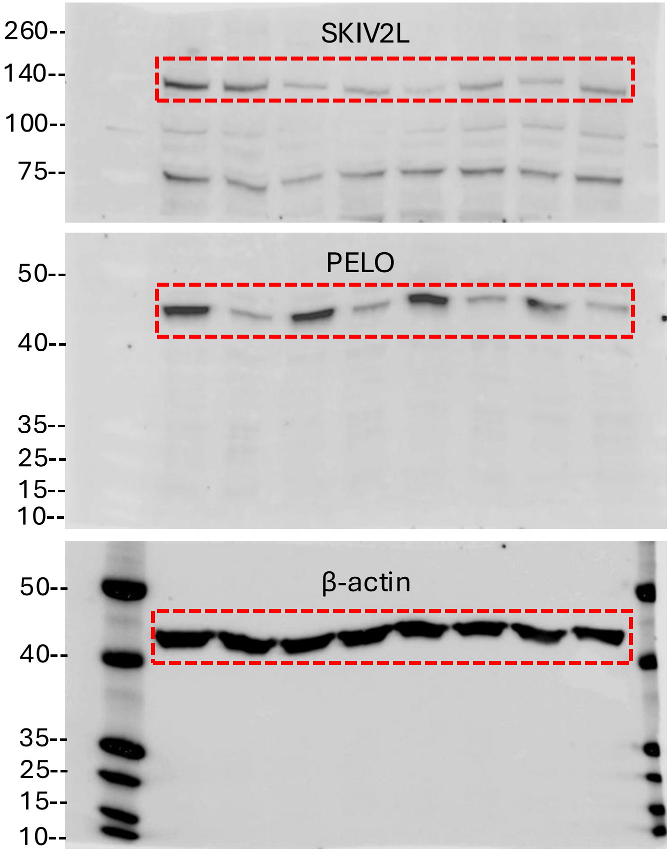

Supplementary Figure 1

Used in Ext Figure 5h

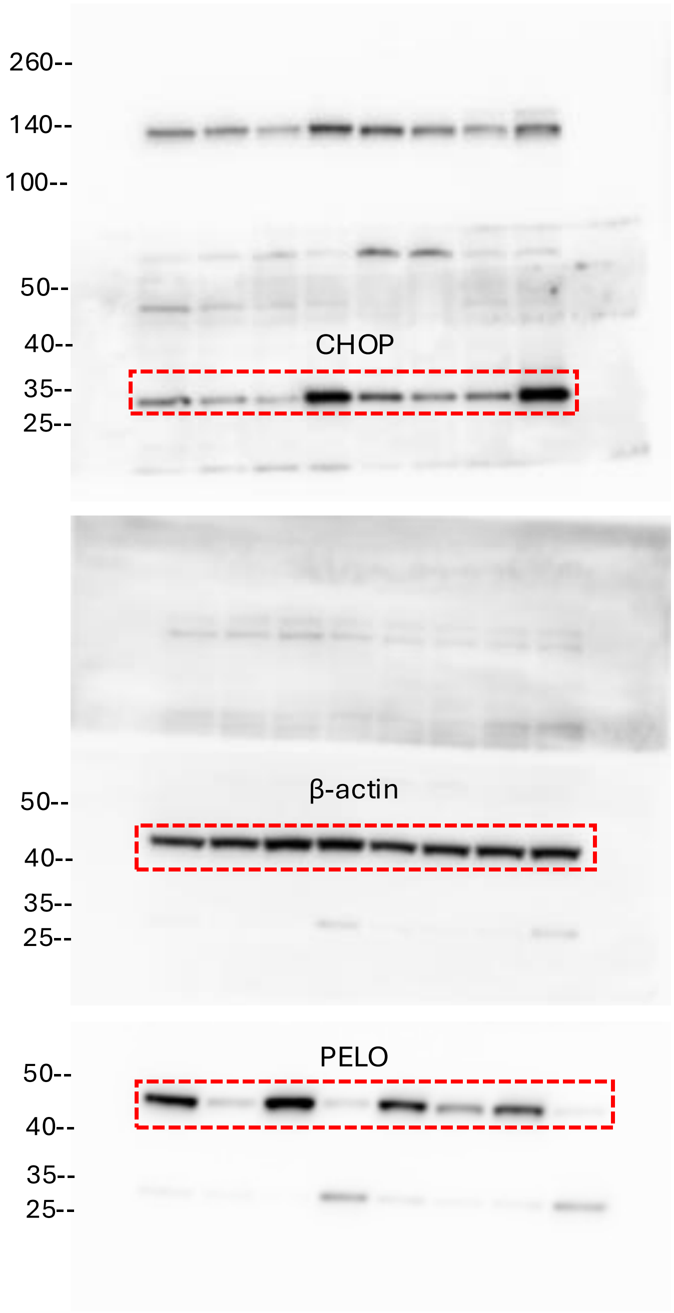

Supplementary Table S1		
**Cell Line:**	**Media Condition:**	**Source**
MIA PaCa-2	DMEM - 4.5g/l D-glucose, L-glutamine, 110mg/L sodium pyruvate (Gibco, 11995073 or Corning, 10-013CV)	ATCC
KM12	RPMI1640 - L-glutamine (Corning, 10-040-CV)	JCRB
DLD1	RPMI1640 (Corning, 10-040-CV)	Academic: Schvartzman's Lab
HCT 116	McCoy’s 5A - L-glutamine (Gibco, 16600082)	ATCC
KP4	DMEM/F12 - L-glutamine, 2.43 g/L sodium bicarbonate (Gibco, 11320033)	RIKEN
293T	DMEM (Gibco; 11995073); 10% FBS ONLY; no PSG	ATCC
SW620	Leibovitz's L-15 - L-glutamine (Gibco, 11415-064) 10% FBS	ATCC
SW837	RPMI-1640 - L glutamine (Corning, 10-040-CV) 10% FBS	ATCC
SF295	RPMI-1640 - L glutamine (Corning, 10-040-CV) 10% FBS	Academic lab: NCI/DCTD
GB1	DMEM - 4.5g/l D-glucose, L-glutamine, 110mg/L sodium pyruvate (Gibco, 11995073 or Corning, 10-013CV)	HSRRB
SU.86.86	RPMI-1640 - L glutamine (Corning, 10-040-CV) 10% FBS	ATCC
IGROV-1	RPMI-1640 - L glutamine (Corning, 10-040-CV) 10% FBS	Academic lab: NCI/DCTD
	**All media was supplemented with:**	
	PSG (penicillin 100 μg ml−1, streptomycin 100 μg ml−1, and l-glutamine 292 μg ml−1; Corning, 30-009-Cl)	
	10% FBS (Gemini Bio-products, 100-106, or Sigma, F4135)	

Supplementary Table S2		
**<u>gRNA</u>**	**<u>CRISPR KO or CRISPRi</u>**	**<u>Sequence</u>**
gRNA Ch2-2	CRISPRi	GGTGTGCGTATGAAGCAGTG
gRNA PELO#1	CRISPRi	CGCTGCGTCTAACAGGCTCG
gRNA PELO#2	CRISPRi	TGCCAGCGGGAACTGTGTAG
gRNA PELO#8	CRISPRi	CCGCTGGCAGCACGCAACAG
gRNA POLR2D	CRISPRi	CGGCGGGTGGCAGCGATCCG
gRNA SF3B1	CRISPRi	AAGATCGCCAAGACTCACGA
gRNA Ch2	AsCas12a CRISPR KO	GTAAAACCCTACTAATATTCCAC
gRNA FOCAD#2	AsCas12a CRISPR KO	AATCACCCCAACTAACCTCCAGG
gRNA FOCAD#5	AsCas12a CRISPR KO	AAGTGGAGCGAATGCAAACAACC
gRNA SKIV2L#1	AsCas12a CRISPR KO	GCCTCACGTACACTATGATCCTC
gRNA SKIV2L#2	AsCas12a CRISPR KO	TTGCTGGACTCCCGAGGAGCCTT
gRNA SKIV2L#5	AsCas12a CRISPR KO	TCTCTCCCATAGTGGGCATTTGA
gRNA TTC37#6	AsCas12a CRISPR KO	AAATAGTATAGTCCTCGCCTAAG
gRNA TTC37#9	AsCas12a CRISPR KO	GTTGTTACAGACTGCAGAAGACC

Supplementary Table S3		
**Antibody**	**Brand/Catalog Number**	**Dilution**
PELO	Abcam 140615	1:500
FOCAD	Novus NBP2-49163	1:500
FOCAD	Millipore Sigma HPA055015	1:500
Vinculin	ThermoFisher Scientific MA5-11690	1:4000
GAPDH	Cell Signaling Technology, 5174S	1:2000
TTC37 “Ski3”	Proteintech 24594-1-AP	1:500
SKIV2L	Proteintech 11462-1-AP	1:1000
phospho-p38 (Thr180/Tyr182)	Cell Signaling Technology 9211S	1:1000
total-p38	Cell Signaling Technology 9212S	1:1000
phospho-JNK/SAPK (Thr183-Tyr185)	Cell Signaling techonology 4668T	1:1000
total-JNK/SAPK	Cell Signaling Technology 9252S	1:1000
alpha-TUBULIN	Millipore Sigma T6793	1:4000
β-Actin	Milipore Sigma, A5441	1:2000
Vinculin	Proteintech, 66305-1-Ig	1:2000
DDIT3/CHOP	Abcam, ab11419	1:1000
DDIT3/CHOP	ABclonal, A21902	1:500

**Protein Ladder**	**Brand/Catalog Number**
Spectra™ Multicolor Broad Range Protein Ladder	Thermo Scientific - 26634
Precision Plus Protein Dual Color Standards	Bio-Rad - 1610374

Supplementary Table S4	
**Plasmid:**	**Addgene Deposit Number:**
pRDA355_sgCiPELO #2	229020
pRDA355_sgCiCh2-2	229021
pRDA355_sgCiPOLR2D	229022
pRDA355H_sgCiPELO #2	228430
pRDA355H_sgCiCh2-2	228431
pRDA355H_sgCiPOLR2D	228432
pLenti_Puro_T2A_Firefly Luciferase	228433
pLenti_Puro_T2A_TTC37	228434
pRDA052H_sgCh2	229023
pRDA052H_sgFOCAD #2	229024
pRDA052H_sgTTC37 #6	228437
pRDA052H_sgSKIV2L #5	228438
pLX313_FOCAD	229025
pRDA052H_sgSKIV2L #1	228440
pRDA052H_sgSKIV2L #2	228441
pRDA052H_sgTTC37 #9	228442
pRDA_052_HygroR	228937
pXPR_051_dCas9-KRAB-MeCP2	228936
pXPR_051d_sgCiChr2-2	228940
pXPR_051d_sgCiPELO #1	229017
pXPR_051d_ sgCiPELO #2	229018
pXPR_023_dCas9-KRAB	228931
pXPR_023d sgCiPELO #1	228932
pXPR_023d sgCiPELO #2	228933
pXPR_023d sgCiPELO #8	228934
pRDA_174_neoR	228935
pLX313 PELO	228938
