# Supplementary material for: SKI complex loss renders 9p21.3-deleted or MSI-H cancers dependent on PELO
Source: Nature. 2025 Feb 5;638(8052):1104–11. doi: 10.1038/s41586-024-08509-3 (PMC11864980; doi:10.1038/s41586-024-08509-3)
Supplement: Supplementary file 2 — Reporting Summary [file 41586_2024_8509_MOESM2_ESM.pdf]

nature portfolio

Corresponding author(s): Edmond Chan, Francisca Vazquez

Last updated by author(s): Oct 26, 2024

Reporting Summary

Nature Portfolio wishes to improve the reproducibility of the work that we publish. This form provides structure for consistency and transparency in reporting. For further information on Nature Portfolio policies, see our Editorial Policies and the Editorial Policy Checklist.

Statistics

For all statistical analyses, confirm that the following items are present in the figure legend, table legend, main text, or Methods section.

n/a	Confirmed	
☐	☒	The exact sample size (*n*) for each experimental group/condition, given as a discrete number and unit of measurement
☐	☒	A statement on whether measurements were taken from distinct samples or whether the same sample was measured repeatedly
☐	☒	The statistical test(s) used AND whether they are one- or two-sided<br>*Only common tests should be described solely by name; describe more complex techniques in the Methods section.*
☐	☒	A description of all covariates tested
☐	☒	A description of any assumptions or corrections, such as tests of normality and adjustment for multiple comparisons
☐	☒	A full description of the statistical parameters including central tendency (e.g. means) or other basic estimates (e.g. regression coefficient) AND variation (e.g. standard deviation) or associated estimates of uncertainty (e.g. confidence intervals)
☐	☒	For null hypothesis testing, the test statistic (e.g. *F*, *t*, *r*) with confidence intervals, effect sizes, degrees of freedom and *P* value noted<br>*Give P values as exact values whenever suitable.*
☒	☐	For Bayesian analysis, information on the choice of priors and Markov chain Monte Carlo settings
☐	☒	For hierarchical and complex designs, identification of the appropriate level for tests and full reporting of outcomes
☐	☒	Estimates of effect sizes (e.g. Cohen's *d*, Pearson's *r*), indicating how they were calculated

Our web collection on statistics for biologists contains articles on many of the points above.

Software and code

Policy information about availability of computer code

Data collection	Immunoblot fluorescence signal was detected with the Li-COR Odyssey CLx and processed with Image Studio (version 5.2.5) software.<br>Immunoblot chemiluminescent signal was detected with the Syngene PXi or Amersham Imager TM 600 and processed with the GeneSys image acquisition software (v1.5.7.0) or ImageJ (version 1.53k).<br>Cell-Titer Glo luminescence signals were detected by the Perkin Elmer Envision Multimode plate reader with version 1.13.3009.1401 of its software. gRNA sequencing was performed on Illumina HiSeq2500. Bulk RNA-seq was sequenced using Illumina NextSeq2000. RT-qPCR was performed using QuantStudio7 Flex Real-Time PCR System (Applied Biosystem).
Data analysis	1. Code used for analysis can be found at https://github.com/broadinstitute/PELO-manuscript.<br>2. We used MSIsensor2 to score microsatellite instability from WGS/WES samples without a matched normal. The codebase for this algorithm can be found at https://github.com/niu-lab/msisensor2.<br>3. The WDL for running the MSIsensor2 workflow can be found in the DepMap Omics repo (https://github.com/broadinstitute/depmap_omics/blob/master/WGS_pipeline/msisensor2.wdl).<br>4. The WDL for running the aggregate microsatellite repeats workflow can be found in the DepMap Omics repo (https://github.com/broadinstitute/depmap_omics/blob/master/WGS_pipeline/aggregate_microsatellite_repeats.wdl).<br>5. WB images were quantified using ImageJ software (version 1.53k)

For manuscripts utilizing custom algorithms or software that are central to the research but not yet described in published literature, software must be made available to editors and reviewers. We strongly encourage code deposition in a community repository (e.g. GitHub). See the Nature Portfolio guidelines for submitting code & software for further information.

Data

Policy information about availability of data

All manuscripts must include a data availability statement. This statement should provide the following information, where applicable:

- Accession codes, unique identifiers, or web links for publicly available datasets
- A description of any restrictions on data availability
- For clinical datasets or third party data, please ensure that the statement adheres to our policy

1. We used CRISPR and genomics data from the DepMap 23Q4 release, including the CRISPRGeneEffect (genetic dependency), OmicsCNSegmentsProfile (segment-level copy number), OmicsExpressionProteinCodingGenesTPMLogp1 (gene-level mRNA expression), OmicsExpressionTranscriptsTPMLogp1Profile (transcript-level mRNA expression), and Model (cell line metadata) files from the figshare repo (https://figshare.com/articles/dataset/DepMap_23Q4_Public/24667905). Additionally, CCLE proteomics data was obtained from the DepMap Portal (https://depmap.org/portal/).
2. Alteration frequencies for FOCAD:HOMDEL, CDKN2A:HOMDEL, MTAP:HOMDEL, and EGFR:MUT_DRIVER in TCGA were obtained from cBioPortal's TCGA PanCancer Atlas 2018.
3. DepMap 23Q4 data for Figs. 1b-f, 3d-e, 4a, and Extended Data Figs. 1a-b, 3d-f,i, 4a-b, 5d-e can be found on figshare at https://plus.figshare.com/articles/dataset/DepMap_23Q4_Public/24667905. The DepMap 24Q2 OmicsSignatures file used in Figs. 1e-d, 3e and Extended Data 1b, 3d-e can be found on figshare at https://plus.figshare.com/articles/dataset/DepMap_24Q2_Public/25880521. CCLE proteomics data shown in Figs. 4a and Extended Data Fig. 3d-e, 4a-b, 5d-e can be found in Nusinow et al. 2020.37 All other data for Figs. 1b-e, 3a,d-e, 4a,c-d and Extended Data Figs. 1a-b, 3a,d-f,i, 4a-b, 5d-g can be found on figshare at [link to be added upon acceptance].

Research involving human participants, their data, or biological material

Policy information about studies with human participants or human data. See also policy information about sex, gender (identity/presentation), and sexual orientation and race, ethnicity and racism.

Reporting on sex and gender	Sex of the individual samples were not collected for the cell lines included in this study.
Reporting on race, ethnicity, or other socially relevant groupings	Race, ethnicity, and other socially relevant groups were not collected for the purposes of this study.
Population characteristics	The population characteristics of cell lines used in this study was not collected.
Recruitment	Patients undergoing clinical care at the Gastrointestinal Cancer Center at Dana-Farber Cancer Institute are routinely screened for enrollment to generate living cancer models from any residual tumor available from clinical procedures (e.g. surgical resection with curative intent). To ensure representation across genders, age, and racial groups, nearly all patients at the Dana-Farber Cancer Center are approached in the pre-operative center for enrollment. We do not anticipate any biases beyond those inherent in the patient population at Dana-Farber Cancer Institute.
Ethics oversight	All human research protocols were conducted under a Dana-Farber Cancer Institute Institutional Review Board (IRB) approved protocol.

Note that full information on the approval of the study protocol must also be provided in the manuscript.

Field-specific reporting

Please select the one below that is the best fit for your research. If you are not sure, read the appropriate sections before making your selection.

☒ Life sciences ☐ Behavioural & social sciences ☐ Ecological, evolutionary & environmental sciences

For a reference copy of the document with all sections, see nature.com/documents/nr-reporting-summary-flat.pdf

Life sciences study design

All studies must disclose on these points even when the disclosure is negative.

Sample size	All cell lines included in the functional genomic datasets were analyzed. No sample size calculation was performed. The differential PELO dependency for the analyses between 9p21.3-/- versus 9p21.3+ and MSI-H versus MSS were highly significant in combined analysis of two independent genomic datasets, demonstrating that this sample size was sufficient. We validated these results using a smaller sample of these cell lines (four cell lines classified as MSS and 9p21.3+ or short 9p21.3 deleted, three 9p21.3-/- cell lines, and three MSI-H cell lines) to confirm that these differences were also present using a single-gene knockdown approach. Three to Four cell lines per group was more than sufficient for this purpose.
Data exclusions	For in vivo growth measurements, mice were inoculated on bilateral flanks with MIA PaCa-2 cells. Mice were randomized once one tumor reached size criteria for randomization, however if the contralateral tumor remained under 50mm3, the smaller tumor was omitted from our study. This was a pre-established exclusion criteria.

Replication	With the exception of the 9p21.3 CRISPR/enAs Cas12a screen (which was performed with two biological replicates), Bulk RNA-seq (which was performed with three biological replicates), the validation for PELO dependency with organoids (performed with 2-3 biological replicates) and WB from Fig.4f and Extended Data Fig 4c-d all experiments were replicated at least once. All attempts at replication successfully confirmed our findings.
Randomization	Once engrafted tumors grew to approximately 150 mm3 in volume, mice were randomized to a dox-containing or standard diet by coin flip.
Blinding	Blinding was not feasible in this study. Knowledge of the tested cell lines were necessary to technically perform the experiment given different culture conditions and different transduction efficiencies. Moreover, the morphology of the cells under microscopy would be telling. In vivo tumor experimentation was also not blinded.

Reporting for specific materials, systems and methods

We require information from authors about some types of materials, experimental systems and methods used in many studies. Here, indicate whether each material, system or method listed is relevant to your study. If you are not sure if a list item applies to your research, read the appropriate section before selecting a response.

Materials & experimental systems

n/a	Involved in the study
☐	☒ Antibodies
☐	☒ Eukaryotic cell lines
☒	☐ Palaeontology and archaeology
☐	☒ Animals and other organisms
☒	☐ Clinical data
☒	☐ Dual use research of concern
☒	☐ Plants

Methods

n/a	Involved in the study
☒	☐ ChIP-seq
☒	☐ Flow cytometry
☒	☐ MRI-based neuroimaging

Antibodies

Antibodies used	PELO: Abcam 140615<br>FOCAD: Novus NBP2-49163 or Millipore Sigma HPA055015<br>Vinculin: ThermoFisher Scientific MA5-11690<br>GAPDH: Cell Signaling Technology 5174S<br>TTC37 “Ski3”: Proteintech 24594-1-AP<br>SKIV2L: Proteintech 11462-1-AP<br>phospho-p38: (Thr180-Tyr182) Cell Signaling Technology 9211S<br>total-p38: Cell Signaling Technology 9212S<br>phospho-JNK/SAPK: (Thr183/Tyr185) Cell Signaling techonology 4668T<br>total-JNK/SAPK: Cell Signaling Technology 9252S<br>alpha-tubulin: Millipore Sigma T6793<br>DDIT3/CHOP Abcam, ab11419 or ABclonal, A21902<br>β-Actin: Milipore Sigma, A5441<br>Vinculin: Proteintech, 66305-1-Ig
Validation	PELO, FOCAD, TTC37 and SKIV2L antibodies were validated by either knockdown and/or cDNA expression. Other antibodies were selected based on trusted citations. All antibodies in this study functioned as expected for their respective assays. Phospho-JNK and phospho-p38 functioned as expected in the presence or absence of positive controls.

Eukaryotic cell lines

Policy information about cell lines and Sex and Gender in Research

Cell line source(s)	Mia PaCa-2, HCT116, 293T, SW620, SW837, SU.86.86 were obtained from ATCC. KM12 was obtained from JCRB. KP4 was obtained from RIKEN. SF295 and IGROV-1 were obtained from the National Cancer Institute Division of Cancer Treatment and Diagnosis. DLD1 was obtained from Juan-Manuel Schvartzman's lab.<br>CCLF_CORE_0001, PANFR0071, and PANFR0127 were obtained from patients with informed consent, at the Dana-Farber Cancer Institute (DFCI).
Authentication	Short-tandem repeat profiling was routinely performed and confirmed the identity of non-organoid cell lines.
Mycoplasma contamination	Mycoplasma testing was routinely performed in all cell lines and were negative for mycoplasma contamination.
Commonly misidentified lines<br>(See ICLAC register)	DepMap performed fingerprinting to ensure the identify of the screened cell lines. All misidentified cell lines were removed from the final dataset.<br>No cell lines used in this study were on the ICLAC register version 12 (released 16 January 2023).

Animals and other research organisms

Policy information about studies involving animals; ARRIVE guidelines recommended for reporting animal research, and Sex and Gender in Research

Laboratory animals	Approximately 6 week old female homozygous NU/J mice obtained from Jackson Laboratories (Catalog 002019) were engrafted with cell lines.
Wild animals	This study did not involve wild animals.
Reporting on sex	No sex-based analysis were performed in this study.
Field-collected samples	This study did not include field-collected samples.
Ethics oversight	Animal work in this manuscript were performed in accordance with the Columbia University Institutional Animal Care and Use Committee (IACUC) animal protocol AC-AABT8654.

Note that full information on the approval of the study protocol must also be provided in the manuscript.

Plants

Seed stocks	No plants were used in this study.
Novel plant genotypes	No plants were used in this study.
Authentication	No plants were used in this study.
